# Time-resolved imaging of contrast kinetics magnetic resonance angiography for the assessment of vascular characteristics in intracranial and extracranial tumors in veterinary patients

**DOI:** 10.3389/fvets.2025.1579582

**Published:** 2025-05-21

**Authors:** Sunghwa Hong, Soyeon Kim, Junghee Yoon, Jihye Choi

**Affiliations:** ^1^Department of Veterinary Medical Imaging, College of Veterinary Medicine, Seoul National University, Seoul, Republic of Korea; ^2^Department of Veterinary Radiology, NEL Animal Medical Center, Anyang-si, Republic of Korea

**Keywords:** dog, CAT, angiography, computed tomography, MRI

## Abstract

**Introduction:**

This study aimed to compare the imaging characteristics and diagnostic utility of Time-Resolved Imaging of Contrast Kinetics (TRICKS) Magnetic Resonance Angiography (MRA) and Computed Tomography Angiography (CTA) for assessing intracranial and extracranial vascular structures in veterinary patients.

**Methods:**

This retrospective methods comparison study included nine client-owned dogs and one cat with head and neck tumors, all of which underwent both CTA and TRICKS MRA. A qualitative assessment of major intracranial and extracranial vessels, as well as tumorassociated vessels—including feeding and draining vessels—was performed. In addition, the signal intensity (SI) of the vessels was quantitatively measured.

**Results:**

Both imaging techniques provided similar SI measurements, although CTA demonstrated significantly higher SI in the basilar artery. CTA also offered higher visibility and clearer delineation of certain arteries and veins, with a significantly shorter acquisition time. However, TRICKS MRA demonstrated comparable or superior performance in visualizing venous structures and effectively identified tumor-related vessels, showing similar diagnostic performance to CTA in detecting feeding and draining vessels. Additionally, TRICKS exhibited a distinct advantage in differentiating vessels from surrounding bone, effectively reducing bone-related artifacts that can interfere with vascular delineation in CTA.

**Discussion:**

TRICKS MRA is a valuable imaging modality for vascular assessment, particularly for evaluating venous structures and tumor-associated circulation. While CTA remains superior for arterial imaging due to its higher spatial resolution and shorter scan time, TRICKS MRA enables dynamic vascular assessment with reduced dependency on contrast injection timing. These findings suggest that TRICKS MRA could serve as a complementary imaging modality in veterinary neuro-oncology, especially for surgical and radiation therapy planning.

## 1 Introduction

In dogs and cats, various nervous system tumors are commonly observed as age-related diseases. The overall estimated incidence of nervous system tumors based on necropsy studies has been reported as 4.5% in dogs and 1.9% in cats (1, 2). These tumors are broadly classified into intracranial tumors, which are further categorized into primary and secondary tumors. Primary intracranial tumors include meningeal tumors such as meningioma, granular cell tumor, and meningeal sarcoma, neuroepithelial tumors such as astrocytoma, oligodendroglioma, choroid plexus tumor, ependymoma, primitive neuroectodermal tumor, and medulloblastoma, and hemopoietic tumors. Secondary intracranial tumors arise from pituitary tumors, nasal tumors, skull tumors, cranial nerve tumors, or metastatic tumors (3). Among primary brain tumors, approximately 90% are classified as meningiomas (45%), gliomas (40%), and choroid plexus tumors (5%), although the reported distribution varies across studies (4).

Before the advancement of diagnostic imaging techniques, the detection of brain tumors in veterinary patients was often delayed, and treatment options were primarily limited to palliative care. However, with recent progress in imaging technology and the integration of artificial intelligence, the accuracy and speed of lesion detection have greatly improved, allowing for more precise diagnoses and the development of tailored treatment plans (5, 6). As imaging modalities continue to evolve, the ability to diagnose intracranial tumors has advanced significantly, leading to growing interest in more proactive and therapeutic interventions.

In particular, craniotomies have become a more widely utilized surgical approach for tumor excision in veterinary neurosurgery (7). Successful surgical outcomes depend on precise preoperative planning, which requires a comprehensive understanding of the vascular anatomy surrounding the tumor to minimize intraoperative risks and complications (8).

Angiographic imaging provides a crucial tool for mapping these structures, enabling to anticipate challenges related to vascular involvement. In human medicine, a thorough understanding of the arterial supply to meningiomas is considered crucial, as this knowledge not only aids in accurate diagnosis but also guides treatment strategies, improving outcomes by enabling targeted management of feeding vessels during surgical or therapeutic procedures (9).

Digital subtraction angiography (DSA) is widely regarded as the gold standard for brain angiography, given its dual role in both diagnosis and, in certain cases, treatment of degenerative diseases, intracranial hemorrhage, and intracranial tumors (10). However, its principal drawbacks include invasiveness, reliance on ionizing radiation, and the need for iodinated contrast agents, which carry a risk of nephrotoxicity (11). CT angiography (CTA) can produce high-quality images with enhanced temporal and spatial resolution and provide vascular assessments comparable to DSA. However, like DSA, CTA requires iodinated contrast agents and exposes patients to ionizing radiation (12).

Magnetic resonance imaging (MRI) is a cornerstone in veterinary diagnostic imaging, offering detailed anatomical and functional insights across a range of clinical applications. Among its techniques, magnetic resonance angiography (MRA) is useful for evaluating vascular anatomy and pathology, particularly in regions requiring precise localization of blood vessels, such as the cranial region. MRA enables accurate mapping of a tumor and surrounding vasculature, clear visualizing of tumor-feeding arteries and nearby critical vascular structures, especially in highly vascularized tumors (13).

MRA is broadly classified into two major categories: non-contrast MRA, which does not require contrast agents, and contrast-enhanced MRA, which uses gadolinium-based contrast agents known to exhibit lower nephrotoxicity compared to iodinated agents (14). Over time, various specialized sequences have been developed to optimize image quality and diagnostic utility in both techniques. However, traditional MRA techniques, such as time-of-flight MRA, are limited by long imaging times that restrict anatomic coverage. While CTA and conventional contrast-enhanced MRA provide broader coverage, they lack dynamic vascular information and depend on precise timing for optimal arterial visualization. Time-resolved imaging of contrast kinetics (TRICKS) overcomes these limitations by capturing dynamic vascular changes with high temporal and spatial resolution. TRICKS enables enhanced visualization of both arterial and venous phases, reducing artifacts, and improving anatomic detail. TRICKS provides superior visualization of vascular structures and enhances the characterization of blood vessels and their relationships to surrounding structures. The anatomical definition of vascular structures associated with intracranial tumors, whether achieved through MRA or contrast-enhanced CT, provides essential information for surgical planning and interventional procedures such as tumor embolization and the local delivery of therapeutic agents (15, 16).

In the previous study, TRICKS has been compared with non-contrast time-of-flight and phase-contrast MRA and TRICKS provided substantially enhanced visualization in both cerebral arteriograms and venograms in dogs (17). However, these findings were derived solely from healthy Beagles, leaving it unclear whether TRICKS would demonstrate similar visualization quality or offer additional diagnostic benefits in animal patients of various breeds with structural pathologies. To date, there is no literature on the application of the TRICKS sequence to tumor-bearing patients in veterinary medicine.

Therefore, this retrospective study examined dogs and cats with intracranial or extracranial structural pathologies and included TRICKS imaging before radiation therapy (RT) in selected patients. This study was performed based on the hypothesis that TRICKS provides vascular visualization comparable to CTA while offering additional diagnostic insights. Furthermore, TRICKS would provide detailed visualization of tumor-feeding and draining vessels in patients who have received RT for head and neck tumors, thereby offering valuable diagnostic information. The purpose of this study is to evaluate the imaging characteristics of the TRICKS sequence in veterinary cranial angiography and to determine its clinical value in characterizing vascular structures in head and neck pathologies, particularly in cases involving neoplastic disease and RT.

## 2 Materials and methods

This study was approved by the Institutional Animal Care and Use Committee of Seoul National University, and the animals were cared for in accordance with the Guidelines for Animal Experiments of Seoul National University (SNU-241218-3).

### 2.1 Case selection

This retrospective methods comparison study included dogs and cats that underwent both CT and MRI between January 2022 and December 2024 at the Seoul National University Veterinary Medical Teaching Hospital. Inclusion criteria were: (1) suspected head and neck tumors, and (2) MRI examinations including TRICKS sequences performed on the same day as CT. Histopathologic confirmation was not a requirement for case selection. The exclusion criteria included cases in which (1) the tumor did not present as a clearly defined solid mass on CT or MRI, or (2) tumor margins were too indistinct to be accurately delineated. All cases were selected through a review of the electronic database by a single doctoral candidate (S.H.H.).

### 2.2 Anesthesia

All CTA and TRICKS MRA scans were performed under general anesthesia. After at least 8 h of fasting, a 22–24 gauges intravenous catheter was placed in the cephalic vein for anesthesia and contrast injection. Anesthesia was induced with an intravascular injection of midazolam (0.2 mg/kg), butorphanol (0.2 mg/kg), and propofol (0.2–0.4 mg/kg). Then, anesthesia was maintained with sevoflurane (Sevofran®, Hana Pharm, Korea) and oxygen (1–1.2 L/min) via an endotracheal tube. During anesthesia, blood pressure was continuously monitored invasively via the pedal artery. Body temperature was manually recorded using a thermometer (MT200, Microlife Corporation, Taipei, Taiwan). During the CTA examination, the CARESCAPE Monitor B650 (GE Healthcare, Helsinki, Finland) was used to monitor oxygen saturation, heart rate, and respiratory rate via ECG tracing and respiratory sensor. In the TRICKS MRA examination, the Datex-Ohmeda Monitor, Type N-MRI 2–00 (GE Healthcare) was used to monitor heart rate and respiratory rate through ECG tracing and respiratory sensor.

### 2.3 CT scan and image post-processing

All patients underwent CT scanning under general anesthesia in ventral recumbency. CT scans were performed using a 160-slice multi-detector CT (Aquilion Lightning 160, Model TSX-036A, Canon Medical Systems, Japan) with a protocol including 120 kVp, 200 mA, a slice thickness of 0.5 mm, and a tube rotation time of 1.0 s. The field of view (FOV) was adjusted according to the size of the patient’s head to include the entire extent of the tumor. Following a pre-contrast CT scan, a test bolus scan was conducted at the level of the common carotid artery and external jugular vein in the neck after injection of 0.5–1 ml/kg of iohexol (Omnipaque 300, GE Healthcare, Oslo, Norway) using a power injector (Medrad Stellant, Bayer HealthCare, Berlin, Germany) at a rate of 2.5–3 ml/s, followed by a 10 ml saline flush. Subsequently, based on the scan delay, arterial and venous-phase CT images were acquired with the administration of 2 ml/kg of iohexol at the same rate of 2.5–3 ml/s, followed by another 10 ml saline flush. The acquired image dataset was reconstructed in transverse, sagittal, and dorsal planes, each with a slice thickness of 0.5 mm and a slice interval of 0.5 mm. The acquisition times for CTA was measured from the start of the pre-contrast phase acquisition to the end of venous phase acquisition.

For post-processing subtraction of CTA, all CT images were transferred to an image analysis software platform (Xelis, Infinitt, South Korea). This software supports a ‘Matched Mask Bone Elimination’ (MMBE) process, which selectively removes bones and calcifications while preserving soft tissue visualization. Upon loading both pre-contrast and post-contrast CT datasets, the software automatically initiated the bone removal process. Using the shape and Hounsfield units (HU) distribution of bony structures in both datasets, the software applied a rigid transformation model to match the volumes accurately. It then selectively eliminated bone structures, while preserving soft tissues and contrast-filled arteries and veins (18–20). The noise reduction filter was set to “none.” The resulting image series were evaluated with a window level of 250 HU and a window width of 550 HU (21). As a reference for image evaluation, maximum intensity projection (MIP) images of the dorsal plane were generated with a slice thickness of 5 mm and an interval of 0.5 mm and adjustments to the window level and width were made as needed for interpretation.

### 2.4 MRA protocol

Following the CT scan, most patients immediately proceeded to MRI in the same position while maintaining anesthesia. MRI examinations were performed using a 1.5-T magnet (SIGNA Creator; GE Healthcare, WI, USA) equipped with an 8-channel coil. An 8-channel knee coil was used for standard diagnostic imaging however an 8-channel flex coil was employed when the knee coil not fully encompass the entire lesion.

TRICKS sequence was acquired to visualize dynamic vascular flow over time with a gadolinium-based contrast agent with the following parameters: the imaging plane was set in the dorsal plane, with a repetition time (TR) of 4–5 ms, and an echo time (TE) of 1–2 ms. The flip angle ranged from 15° to 50° (e.g., 15, 20, 25, 50). The FOV was adjusted from 180 to 280 mm (180, 200, 210, 220, 230, 240, 280), with matrix sizes including 192 × 192, 200 × 200, 220 × 220, 224 × 224, 320 × 224, and 320 × 256. Pixel sizes were 0.9 × 1.1, 0.8 × 1.2, 0.9 × 1.2, 1.1 × 1.1, 0.9 × 1.2, 1.2 × 1.2, 0.8 × 0.8, 1.3 × 1.3, 1 × 1, or 0.9 × 0.9 mm. Slice thickness ranged from 1.6 to 2.6 mm (1.6, 1.8, 2.0, 2.2, 2.6 mm), and the number of excitations (NEX) varied between 0.5 and 1 (0.5, 0.75, 1). The total number of phases captured was between 17 and 40 (17, 25, 30, 40). The scan delay time was generally set to a minimum, although a delay of 10–20 s was applied in some patients.

For TRICKS MRA, a single intravenous dose (0.2 mmol/kg) of a gadolinium-based contrast agent (Dotarem, Guerbet, France) was administered via an automatic power injector (OptiStar® Elite, Mallinckrodt, Missouri, USA) at a rate of 0.2–2 ml/s through a cephalic vein catheter, immediately, followed by 6 ml of normal saline flush at the same rate. During this period, 17–40 frames were captured to visualize the progression of the contrast agent within the blood vessels. The TRICKS acquisition time was defined as the period from the start of post-contrast imaging to the end of the sequence, excluding the baseline mask acquisition. Following subtraction of the baseline images, 3D reconstructed MRA images were generated using maximum intensity projection (MIP), and the images for each time phase were combined into a single series.

### 2.5 Image analysis

All CT and MRI images were transferred to a picture archiving and communication system (Infinitt PACS; Infinitt Healthcare, Seoul, South Korea) for analysis. Images evaluation was performed qualitatively for the overall intracranial and extracranial vascular structures and the tumor-associated feeding and draining vessels and also quantitatively for signal intensity of major artery and vein on CTA and TRICKS.

The qualitative analysis for overall intracranial and extracranial vascular structures was performed by two independent observers, both PhD candidates in veterinary radiology, with one (S.H.H) having over 10 years of experience and the other (S.Y.K) having 5 years of experience in both clinical and academic settings. The qualitative analysis for tumor-associated feeding and draining vessels and the quantitative analysis were conducted by a single observer (S.H.H). Although the observers were not completely blinded to the technique because of inherent differences in the signal characteristics of the CTA and MRA, CTA and TRICKS images were presented to the observers in a randomized order. The two observers analyzed the images in reverse patient order and were blinded to the patient’s information, medical history, and tumor location. Each image was evaluated once, and the data from this single assessment were used for analysis.

### 2.6 Qantitative assessment

The basilar artery and transverse sinus were used for the quantitative assessment. The relative signal intensity (rSI) of these vessels was calculated separately by comparing each vessel with the adjacent soft tissue to evaluate the clarity of vascular visualization and the diagnostic capability of CTA and TRICKS MRA (22–24). Additionally, a combined analysis was performed using the mean rSI values of both vessels to provide an overall comparison of vascular visualization and the diagnostic capability of each imaging technique.

In CTA, the rSI of each vessel was measured using static images in the arterial and venous phases with a window level of 250 Hounsfield Units (HU) and window width of 550 HU.

**Figure 1:**
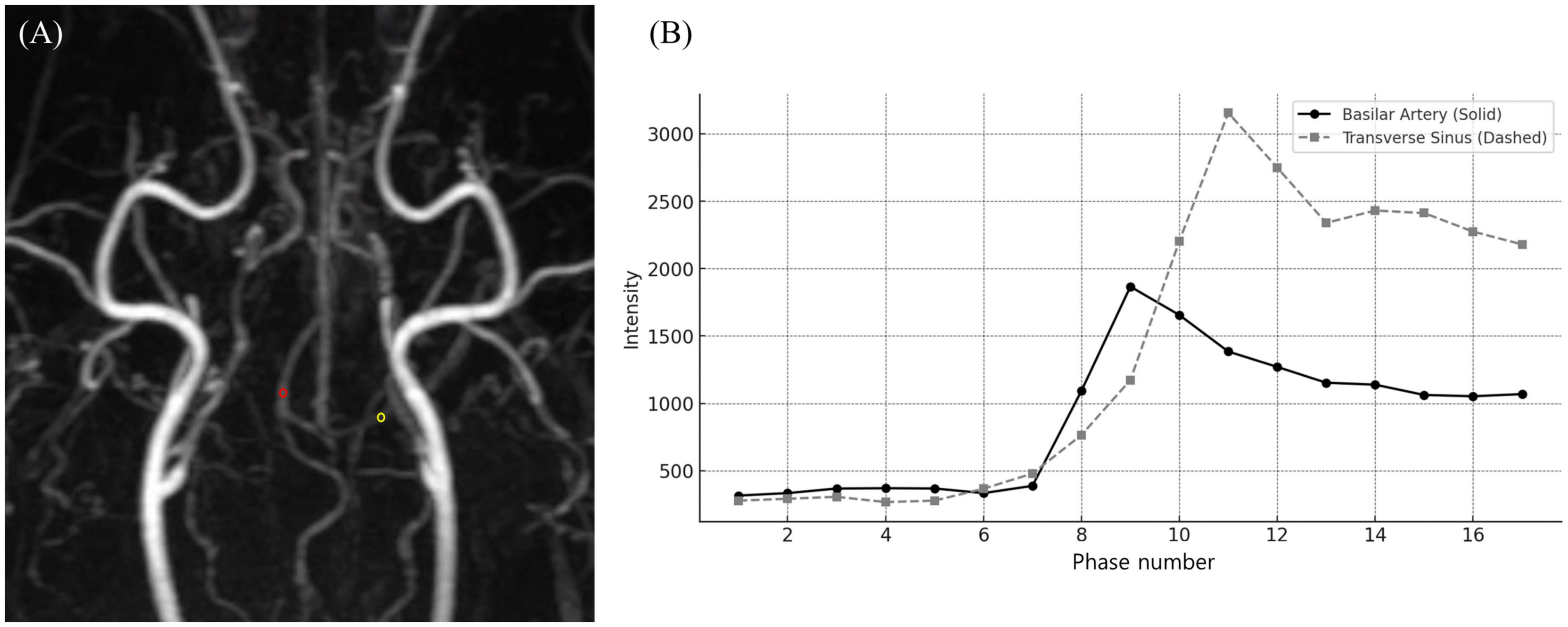
Time-resolved imaging of contrast kinetics (TRICKS) analysis of vascular signal intensity changes. **(A)** A dorsal Maximum intensity projection (MIP) image from the 9th phase of TRICKS sequence, displaying the intracranial vascular structures. The red circle represents the region of interest (ROI) placed on the basilar artery, and the yellow circle marking the ROI on the transverse sinus for signal intensity measurement. **(B)** Time-intensity curve of the basilar artery (solid line) and transverse sinus (dashed line), demonstrating the temporal changes in signal intensity across multiple phases. The basilar artery exhibited its peak signal intensity at phase 9, followed by a gradual decline. Whereas, the transverse sinus reached its highest signal intensity at phase 11, with a subsequent plateau and gradual decline. TRICKS showed the sequential contrast filling patterns of arterial and venous structures for dynamic vascular assessment.

In TRICKS MRA, unlike CTA, ReadyView software (GE Healthcare) was used to generate time-intensity curves (TICs) from time-dependent MIP images across all phases. The phase exhibiting the highest SI for each vessel was identified, and vascular evaluation was performed based on that phase, with the region of interest (ROI) designated for each vessel in the corresponding phase (Figure 1).

Then, for both imaging techniques, the SI of the vessel was measured by placing a circular ROI over the area with the highest signal intensity and the largest diameter, with an ROI size ranging from 1 to 3 mm. The same ROI size was used to measure the SI of adjacent stationary soft tissue, such as the brain or muscle next to the vessel. The rSI was calculated using the following formula:


$$\begin{array}{l} \mathrm{rSI}=(\text{Vessel}\;\mathrm{SI}-\text{Adjacent soft tissue}\;\mathrm{SI})\\ /(\text{Vessel}\;\mathrm{SI}+\text{Adjacent soft tissue}\;\mathrm{SI}) \end{array}$$

Additionally, a combined analysis of the mean rSI values of both vessels was performed to provide an overall comparison of vascular visualization and the diagnostic capability of each imaging technique.

### 2.7 Qualitative assessment

The qualitative assessment was evaluated for the overall intracranial and extracranial vascular structures and the tumor-associated feeding and draining vessels. The CTA images were evaluated primarily using MIP dorsal plane and transverse plane images. For TRICKS images, the time phase previously used for quantitative analysis was primarily assessed, with additional time phases evaluated as needed.

The overall intracranial and extracranial vascular structures were assessed from total 21 vessels in each patient: The intracranial arteries assessed included the basilar artery, rostral cerebral artery, middle cerebral artery, caudal cerebral artery, rostral cerebellar artery, caudal communicating artery, and internal carotid artery. The extracranial arteries evaluated included the infraorbital artery, descending palatine artery, maxillary artery, caudal deep temporal artery, and caudal auricular artery. For the intracranial veins, the evaluated vessels included the dorsal sagittal sinus, transverse sinus, temporal sinus, sigmoid sinus, straight sinus, and dorsal cerebral vein. The extracranial veins assessed included the maxillary vein, superficial temporal vein, and dorsal external ophthalmic vein. For each vessel, five evaluation criteria were scored using a subjective four-point scale system, assessing vascular visibility, vascular delineation, vascular connectivity, artifact associated with adjacent bone, and overall image quality (Table 1). Vascular visibility assessed the signal intensity and uniformity of the vessel morphology. Vascular delineation was evaluated based on the clarity of vessel margins and their conspicuity from the surrounding tissues. Vascular connectivity was assessed the continuity of vessels along their entire course without interruption, evaluating whether the vessels were consistently visualized or were partially obscured by artifacts. Artifact from adjacent bone was evaluated to assess the impact of high-density skull bones on the vascular signal and morphology. Overall image quality was assessed based on the presence and severity of artifacts, the existence of surrounding noise, and their effect on the diagnostic usability of the images.

**Table 1:** Qualitative evaluation criteria for comprehensive intracranial and extracranial vascular structures in CT angiography and time-resolved imaging of contrast kinetics (TRICKS).

Evaluation factor	Scoring criteria
Vascular visibility	1 = The vessel is not visualized, making evaluation impossible. 2 = The vessel is barely distinguishable, with unclear morphological definition. 3 = The vascular morphology is distinguishable, though with slightly reduced signal intensity. 4 = vessel exhibits a high-contrast signal against the surrounding structures, making it clearly distinguishable.
Vascular delineation	1 = The vessel is not visualized, making evaluation impossible. 2 = Vessel delineation is generally irregular. 3 = Vessel delineation has slight irregularities but remains overall acceptable. 4 = Vessel delineation is smooth and complete vessel borders.
Vascular connectivity	1 = The vessel is not visualized, making evaluation difficult. 2 = Less than half of the entire vessel length is observed. 3 = More than half of the entire vessel length is observed, but with discontinuities. 4 = The entire vessel length is fully visualized without interruptions.
Artifact associated with bone	1 = The vessel is mostly obscured by surrounding bone, making evaluation difficult. 2 = Diffuse and inhomogeneous signal changes are observed throughout the vessel due to adjacent bone. 3 = Partial distortion of the vascular morphology due to interference from surrounding bone. 4 = No influence from surrounding bone is observed.
Overall image quality	1 = Severe artifacts, vessels are poorly visualized, and image quality is rated as very poor. 2 = Artifacts and noise are present and affect image evaluation, resulting in fair image quality. 3 = No artifacts are present, though some surrounding noise exists; however, it does not interfere with image evaluation, ensuring good image quality. 4 = No artifacts and noise are present, resulting in excellent image quality.

The tumor associated feeding and draining vessels were evaluated using a 3-point scale for four criteria (Table 2). Tumor-vessel differentiation was determined whether the tumor-associated vessels could be clearly distinguished from the tumor itself. The intratumoral blood flow visualization assessed the ability to differentiate blood flow within the tumor parenchyma. Conspicuity of the vessel from bone was evaluated whether overlying bone structures obscured or interfered the visibility of tumor-associated vessels. Lastly, feeding and draining vessel identification was determined whether both vessel types could be clearly visualized and differentiated. The feeding vessel was defined as the closest arterial vessel supplying the tumor, while the draining vessel was identified as the closest venous vessel showing connectivity to the tumor.

**Table 2:** Qualitative evaluation criteria for tumor-associated feeding and draining vessels in CT angiography and time-resolved imaging of contrast kinetics (TRICKS).

Evaluation factor	Scoring criteria
Tumor-vessel differentiation	1 = Difficult to distinguish 2 = Visible only upon careful inspection 3 = Easily distinguishable
Intratumoral blood flow visualization	1 = Not distinguishable 2 = Visible upon careful inspection 3 = Easily visible
Conspicuity the vessel from bone	1 = Significantly hindered, making differentiation difficult 2 = Somewhat hindered 3 = Not hindered at all
Feeding and draining vessel identification	1 = Difficult to identify either type 2 = Only one vessel type identified 3 = Both feeding and draining vessels identified

The evaluation scores for each vessel were assessed separately in both imaging techniques, allowing for direct vessel-to-vessel comparisons between CTA and TRICKS. Additionally, the scores within each imaging technique were combined to compare the overall performance of CTA and TRICKS.

### 2.8 Statistical analyses

Statistical analysis was performed using SPSS for Windows (version 29.0; SPSS Inc., Chicago, IL, USA). These statistical analyses were conducted under the supervision of a doctoral-level statistician working at a human hospital. The normality of data distribution for quantitative analysis was assessed using the Shapiro–Wilk test. For quantitative assessments, Pearson correlation coefficients and paired t-tests were performed to evaluate mean differences between CTA and TRICKS in the basilar artery and transverse sinus separately, as well as for the combined analysis of both vessels. For qualitative assessments, the evaluation scores were analyzed using different statistical tests based on the comparison type. The overall intracranial and extracranial vascular structures were compared between CTA and TRICKS using both the Mann–Whitney U test and the Wilcoxon signed-rank test for combined scores. The vessel-by-vessel comparisons were conducted exclusively using the Wilcoxon signed-rank test. Additionally, the tumor-associated vessels were also compared using the Wilcoxon signed-rank test. For analyses that included two observers, interobserver agreement was assessed using the intraclass correlation coefficient (ICC), where values below 0.4 indicate poor agreement, values between 0.41 and 0.6 indicate moderate agreement, values from 0.61 to 0.8 indicate good agreement, and values above 0.8 indicate excellent agreement (25). A *p*-value < 0.05 was considered statistically significant for all analyses. Data are reported as mean ± standard deviation (SD).

## 3 Results

A total of 15 patients underwent imaging, of which five were excluded due to poor image quality caused by TRICKS sequence failure, resulting in the final inclusion of imaging data from 10 patients in the study. Among the included patients, there were nine dogs and one cat. The dog breeds included two Poodles and one each of Bull Terrier, Dachshund, Cocker Spaniel, Chihuahua, Spitz, Maltese, and mixed breed dog. The single cat was a Persian. Their ages ranged from 6 to 15 years, with a mean age of 11.5 years. There was one intact female, one intact male, four spayed females, and four castrated males. The body weight ranged from 2.8 to 20 kg, with a mean weight of 11.5 kg. All patients underwent RT with varying protocols for head and neck tumors confirmed via a histopathological examination, including meningioma in four cases, nasal adenocarcinoma in two cases, maxillary squamous cell carcinoma in two cases, and single cases of ceruminous gland adenocarcinoma and nasal fibrosarcoma. CTA and TRICKS MRA examinations were performed one to three times per patients after RT.

All CTA and TRICKS were performed with no complications related to anesthesia or contrast administration in any patient. The acquisition time for CTA averaged 40 ± 6 s (ranging from 33 to 51 s), with additional time required for multiplanar and MIP reconstruction using MMBE. The image acquisition time for TRICKS MRA consisted of a post-contrast image acquisition time. Before the contrast agent injection, a mask acquisition period of averaged 18.06 ± 4.8 s (ranging from 11.3 to 24.5 s) was used to obtain the baseline image. The post-contrast acquisition time had an average duration of 109.2 ± 11.8 s, ranging from 92 to 126 s. Images were acquired at an average interval of 4.2 ± 1.4 s (ranging from 2.3 to 6.1 s). As a result, the total acquisition time averaged 127.3 ± 15 s (ranging from 110.4 to 148.6 s). TRICKS MRA was automatically post-processed, generating phase-specific MIP images.

### 3.1 Quantitative assessment of image quality

The rSI values for both CTA and TRICKS MRA in the basilar artery and transverse sinus followed a normal distribution, with Shapiro–Wilk statistics (W) of 0.914 (*p* = 0.075) for CTA and 0.939 (*p* = 0.229) for TRICKS in the basilar artery, while W values for the transverse sinus were 0.914 (*p* = 0.075) for CTA and 0.939 (*p* = 0.229) for TRICKS. In the Pearson paired-sample correlation coefficients for the relationship between CTA and TRICKS measurements, the rSI values of the basilar artery, transverse sinus and combined analysis are presented in Table 3. There was no statistically significant difference in rSI between CTA and TRICKS when comparing the basilar artery and transverse sinus individually, as well as when analyzing both vessels combined.

**Table 3:** Mean and standard deviation of relative signal intensity (rSI) values, *p*-values, and correlation coefficients (r) for CT angiography (CTA) and Time-resolved imaging of contrast kinetics (TRICKS) in the basilar artery, transverse sinus, and combined analysis.

Vessel	CTA	TRICKS	*p*-value	Correlation (r)
Basilar artery	0.8861 ± 0.1029	0.6502 ± 0.1202	0.524	0.229
Transverse sinus	0.8040 ± 0.1287	0.8734 ± 0.0802	0.469	0.260
Combined (Mean of both vessels)	0.845 ± 0.1210	0.7618 ± 0.1517	0.614	−0.120

*p-value < 0.05.

**Figure 2:**
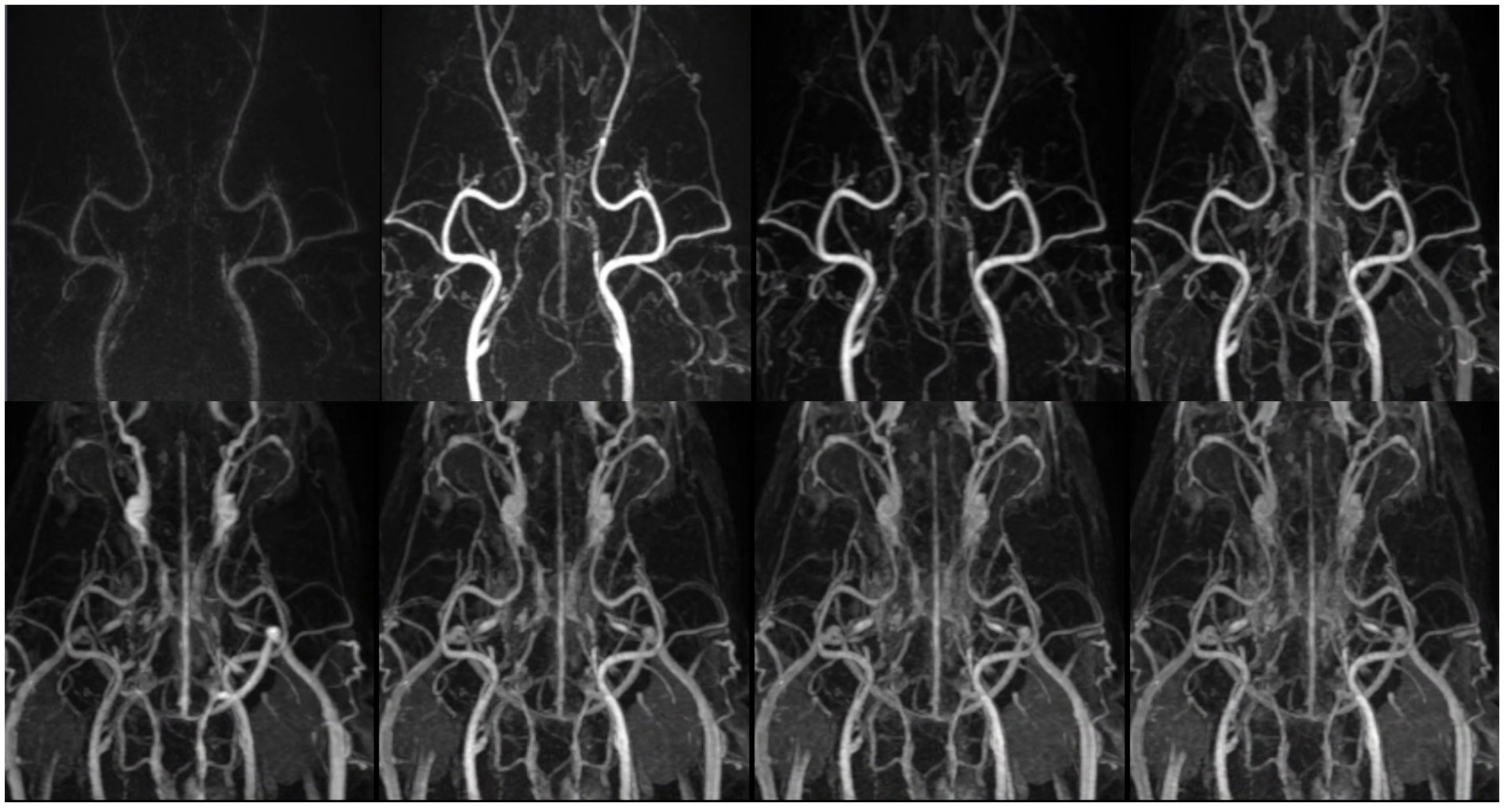
Temporal evolution of contrast enhancement in time-resolved imaging of contrast kinetics (TRICKS). Sequentially collapsed dorsal maximum intensity projection (MIP) images from multiple temporal phases visualize the dynamic contrast filling of intracranial and extracranial vascular structures. The upper row represents the early to mid-arterial phases (7th, 8th, 9th, and 10th temporal phases), demonstrating the progressive enhancement of the major feeding arteries. However, clear distinction between the arterial and venous phases was challenging due to overlapping contrast enhancement. Sufficient contrast filling of the cerebral dural venous sinuses was obtained before the 10th temporal phase. The lower row corresponds to the later phases (11th, 13rd, 15th, and 17th temporal phases), showing venous opacification and the visualization of draining vessels. The temporal resolution was 4.5 s for each phase. TRICKS demonstrated adequate visualization of both arteriograms and venograms.

The rSI differences between CTA and TRICKS were analyzed using paired t-tests, which aim to determine whether there is a statistically significant mean difference between two related measurements, and effect sizes summarized in Table 4. In the basilar artery, CTA showed significantly higher rSI compared to TRICKS, with a large effect size (Cohen’s d = 1.695). However, in the transverse sinus, no significant difference was observed between CTA and TRICKS and the effect size was small (Cohen’s d = −0.523). When the rSI values of both vessels were combined for an overall comparison, no significant difference was found between CTA and TRICKS and the effect size was small (Cohen’s d = 0.406). These results indicate that CTA provides superior signal intensity in arterial structures, whereas in the transverse sinus and combined vessel analysis, no significant difference was found between CTA and TRICKS. The dynamic changes in signal intensity observed in TRICKS MRA, particularly the peak enhancement times of the basilar artery (Phase 9) and transverse sinus (Phase 11), are presented in Figure 2, providing the temporal changes of contrast enhancement in TRICKS.

**Table 4:** Paired T-test and effect size analysis for quantitative analysis of CTA and TRICKS in the basilar artery, transverse sinus, and combined analysis.

Vessel	Mean difference (CTA - TRICKS)	95% Confidence interval (Lower, Upper)	*t*-value	df	*p*-value	Cohen’s d (Effect size)	Effect size interpretation
Basilar artery	0.2359	0.1364, 0.3355	5.360	9	< 0.001	1.695	Large
Transverse sinus	−0.0694	−0.1645, 0.0256	−1.653	9	0.133	−0.523	Small
Combined (Mean of both vessels)	0.0832	−0.0128, 0.1792	1.815	19	0.085	0.406	Small

*p-value < 0.05. CTA, Computed Tomography Angiography; TRICKS, Time-resolved imaging of contrast kinetics; rSI, relative signal intensity; df, degrees of freedom.

### 3.2 Qualitative assessment of image quality

The qualitative assessment was performed for the overall intracranial and extracranial vascular structures as well as the tumor-associated feeding and draining vessels. The ICC analysis for the evaluation of the overall intracranial and extracranial vascular structures demonstrated high inter-rater reliability for all 21 vessels both CTA and TRICKS (Table 5). The mean ICC values for overall assessments were 0.980 [95% confidence interval (CI): 0.977–0.982] for CTA and 0.999 (95% CI: 0.999–0.999) for TRICKS, indicating excellent agreement.

**Table 5:** Intraclass correlation coefficient (ICC) values and 95% confidence intervals of qualitative assessment for overall vascular structures.

Vessel	ICC	95% confidence interval
Basilar artery	1	1
Caudal auricular artery	1	1
Caudal cerebral artery	0.993	0.989–0.995
Caudal communicating artery	0.993	0.989–0.995
Caudal deep temporal artery	0.985	0.978–0.990
Descending palatine artery	1	1
Dorsal cerebral vein	0.995	0.992–0.996
Dorsal external ophthalmic vein	1	1
Dorsal sagittal sinus	1	1
Infraorbital artery	1	1
Internal carotid artery	1	1
Maxillary artery	1	1
Maxillary vein	1	1
Middle cerebral artery	1	1
Rostral cerebellar artery	1	1
Rostral cerebral artery	1	1
Sigmoid sinus	1	1
Straight sinus	0.979	0.968–0.986
Superficial temporal vein	1	1
Temporal sinus	1	1
Transverse sinus	1	1

**Figure 3:**
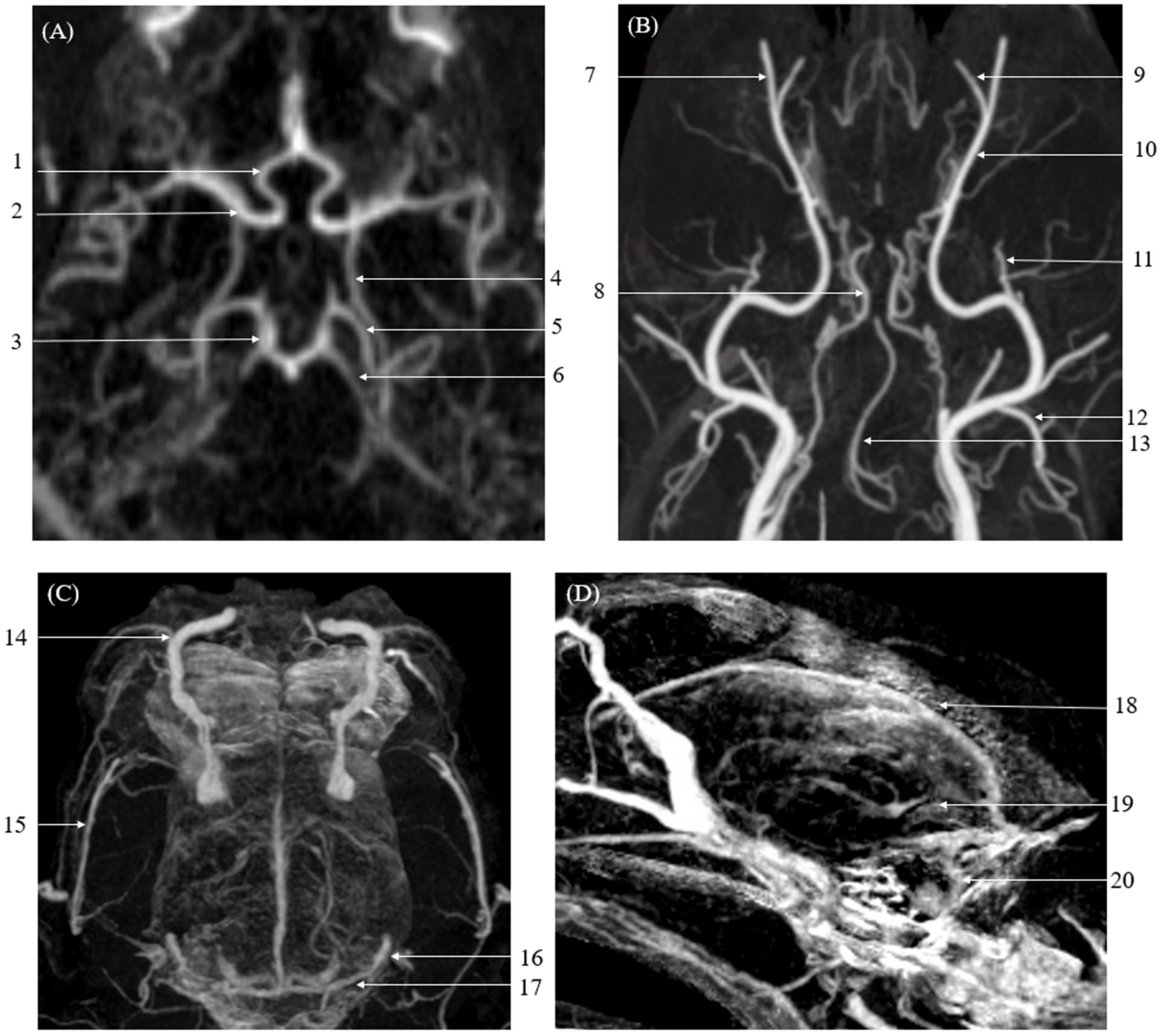
Maximum Intensity Projection (MIP) reconstruction images from a Computed Tomography Angiography (CTA) scan showing various intra-cranial and extra-cranial vessels in a dog. Some of these vessels exhibited significantly higher vascular visibility and delineation compared to Time-Resolved Imaging of Contrast Kinetics (TRICKS). The identified vessels include the basilar artery, caudal auricular artery, caudal cerebral artery, caudal communicating artery, caudal deep temporal artery, descending palatine artery, infraorbital artery, maxillary artery, middle cerebral artery, rostral cerebellar artery, rostral cerebral artery, dorsal sagittal sinus, superficial temporal vein, temporal sinus, and transverse sinus. **(A)** Arterial-phase MIP image at the level of the basilar artery. **(B)** Arterial-phase MIP image displaying arterial vasculature in the dorsal plane. **(C)** Venous-phase MIP image showing venous structures in the dorsal plane. **(D)** Sagittal plane MIP image highlighting venous vasculature. (1 rostral cerebral artery, 2 middle cerebral artery, 3 caudal communicating artery, 4 internal carotid artery, 5 caudal cerebral artery, 6 rostral cerebellar artery, 7 infraorbital artery, 8 internal carotid artery, 9 descending palatine artery, 10 maxillary artery, 11 caudal deep temporal artery, 12 caudal auricular artery, 13 basilar artery, 14 dorsal external ophthalmic vein, 15 superficial temporal vein, 16 temporal sinus, 17 transverse sinus, 18 dorsal sagittal sinus, 19 straight sinus, and 20 sigmoid sinus).

The qualitative evaluation of the overall intracranial and extracranial vascular structures is presented in Table 6. The vascular visibility was significantly higher in CTA than in TRICKS across the overall score distribution. Specifically, CTA demonstrated significantly greater vascular visibility in the basilar artery, caudal auricular artery, caudal cerebral artery, caudal communicating artery, caudal deep temporal artery, descending palatine artery, dorsal sagittal sinus, infraorbital artery, maxillary artery, middle cerebral artery, rostral cerebellar artery, superficial temporal vein, temporal sinus, and transverse sinus (Figure 3). For the remaining vessels, no significant difference was observed between the two modalities.

**Table 6:** Qualitative assessments of the overall intracranial and extracranial vascular structures in CT angiography (CTA) and Time-resolved imaging of contrast kinetics (TRICKS).

Evaluation factors	CTA (*n* = 10)		TRICKS (*n* = 10)	
	Mean ± SD	Median [IQR]	Mean ± SD	Median [IQR]
Vascular visibility	3.59 ± 0.74	4 [3.00–4.00]^ab^	3.37 ± 0.64	3 [3.00–4.00]
Vascular delineation	3.5 ± 0.83	4.00 [3.00–4.00]	3.45 ± 0.71	3.50 [3.00–4.00]
Vascular connectivity	3.63 ± 0.75	4.00 [3.00–4.00]	3.72 ± 0.55	4.00 [3.00–4.00]
Artifact associated with bone	3.53 ± 0.86	3.50 [3.00–4.00]	3.99 ± 0.14	4.00 [4.00–4.00]^ab^
Overall image quality	3.91 ± 0.36	4.00 [4.00–4.00]^ab^	2.99 ± 0.14	3.00 [3.00–3.00]

SD, standard deviation; IQR, Interquartile range. a = Wilcoxon signed-rank test (*p* < 0.05), b = Mann–Whitney U test (*p* < 0.05).

**Figure 4:**
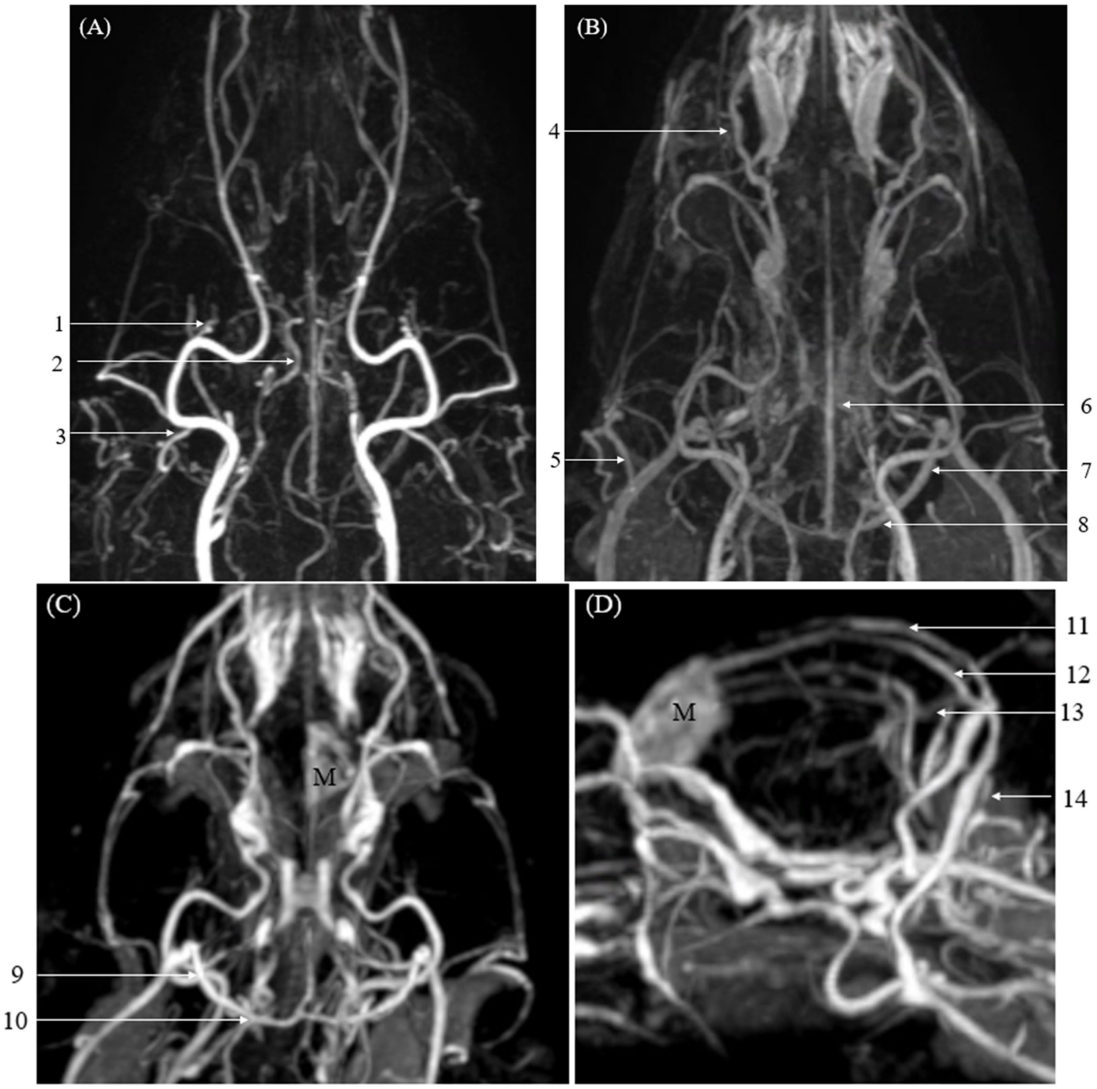
Images of intracranial and extracranial vessels acquired using time-resolved imaging of contrast kinetics (TRICKS) in two dogs. Compared to computed tomography angiography (CTA), TRICKS demonstrated higher delineation in the dorsal cerebral vein, dorsal sagittal sinus, sigmoid sinus, straight sinus, temporal sinus, and transverse sinus, and higher connectivity in the dorsal cerebral vein, dorsal sagittal sinus, straight sinus, and temporal sinus. **(A)** Maximum intensity projection (MIP) reconstruction of the dorsal plane image from the 9th phase of TRICKS in the first dog. **(B)** MIP reconstruction of the dorsal plane image from the 17th phase of TRICKS in the first dog. **(C,D)** MIP reconstruction of the dorsal and sagittal plane images from the 9th phase of TRICKS in the second dog. (1 caudal deep temporal artery, 2 internal carotid artery, 3 caudal auricular artery, 4 dorsal external ophthalmic vein, 5 superficial temporal vein, 6 cerebral sagittal sinus, 7 temporal sinus, 8 transverse sinus, 9 temporal sinus, 10 transverse sinus, 11 cerebral sagittal sinus, 12 dorsal cerebral vein, 13 straight sinus, 14 sigmoid sinus, and M meningioma).

In vascular delineation of the overall vessels, the combined evaluation scores were not significantly different between CTA and TRICKS. However, when evaluated on a vessel-by-vessel basis, CTA exhibited significantly superior vascular delineation in the caudal auricular artery, caudal cerebral artery, caudal deep temporal artery, descending palatine artery, middle cerebral artery, rostral cerebellar artery, and rostral cerebral artery (Figure 3). Conversely, TRICKS demonstrated significantly superior vascular delineation in the dorsal cerebral vein, dorsal sagittal sinus, sigmoid sinus, straight sinus, temporal sinus, and transverse sinus (Figure 4). No significant difference was found in the other vessels.

For vascular connectivity, there was no significant difference between CTA and TRICKS in the overall score distribution. However, in vessel-by-vessel comparisons, TRICKS showed significantly superior connectivity in the dorsal cerebral vein, dorsal sagittal sinus, straight sinus, and temporal sinus to TRICKS, while no significant differences were observed in the other vessels (Figure 4).

**Figure 5:**
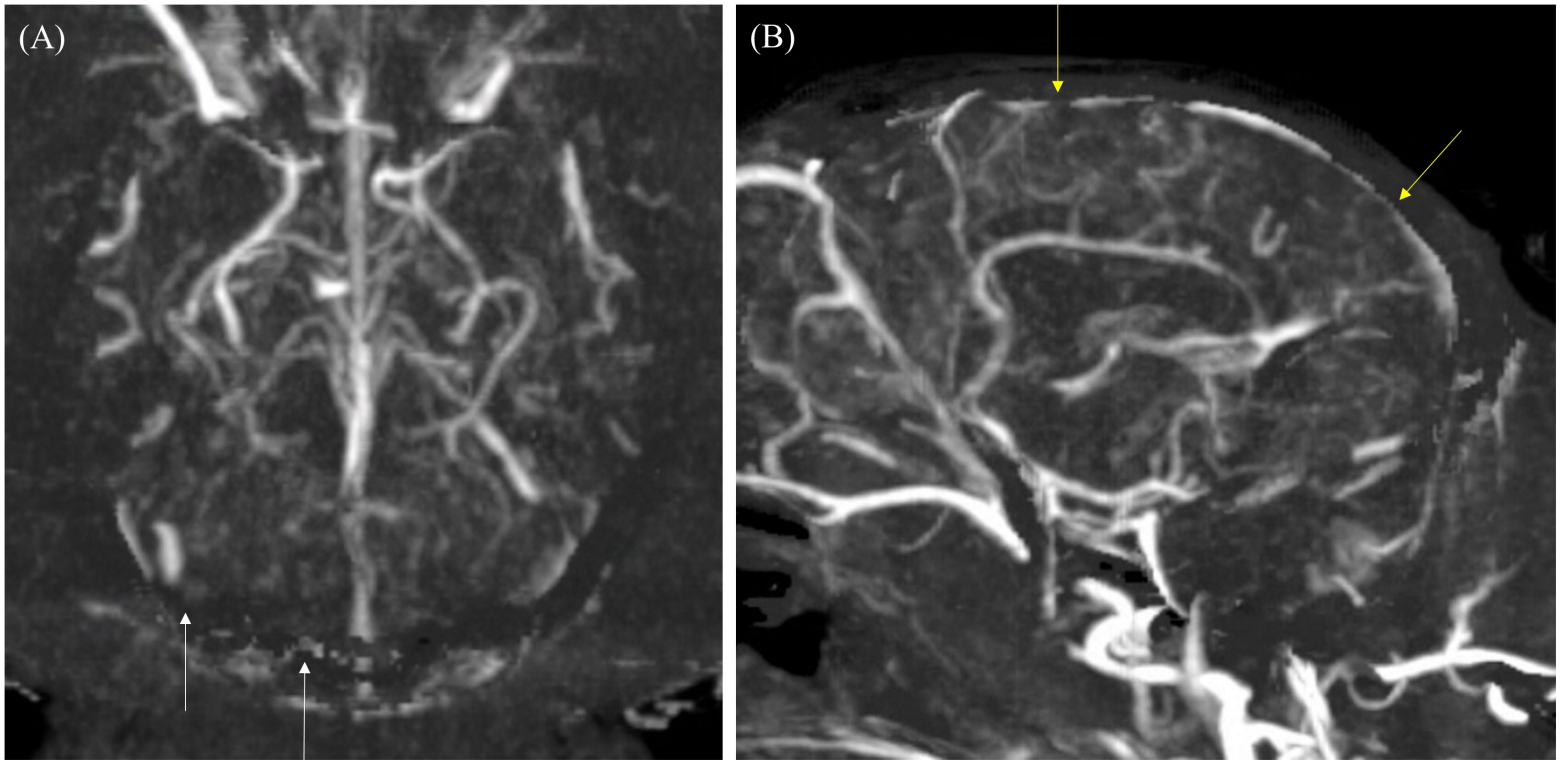
Maximum intensity projection (MIP) reconstruction images of the dorsal and sagittal planes from a computed tomography angiography (CTA) scan in a dog. **(A)** In the dorsal plane, the transverse sinus and temporal sinus (white arrows) appear poorly delineated due to artifacts from bone subtraction. **(B)** In the sagittal plane, the cerebral sagittal sinus (yellow arrow) is partially visible with irregular margins due to artifacts from bone subtraction.

Regarding artifacts associated with bone, TRICKS demonstrated significantly fewer artifacts than CTA in both the overall score distribution and the vessel-by-vessel comparisons. This result was particularly evident in the dorsal cerebral vein, dorsal sagittal sinus, sigmoid sinus, straight sinus, temporal sinus, and transverse sinus, where TRICKS exhibited significantly reduced artifacts compared to CTA (Figure 5).

However, in the overall image quality, CTA was found to be significantly superior to TRICKS in both the overall score distribution and vessel-by-vessel comparisons, demonstrating consistently higher image quality.

**Figure 6:**
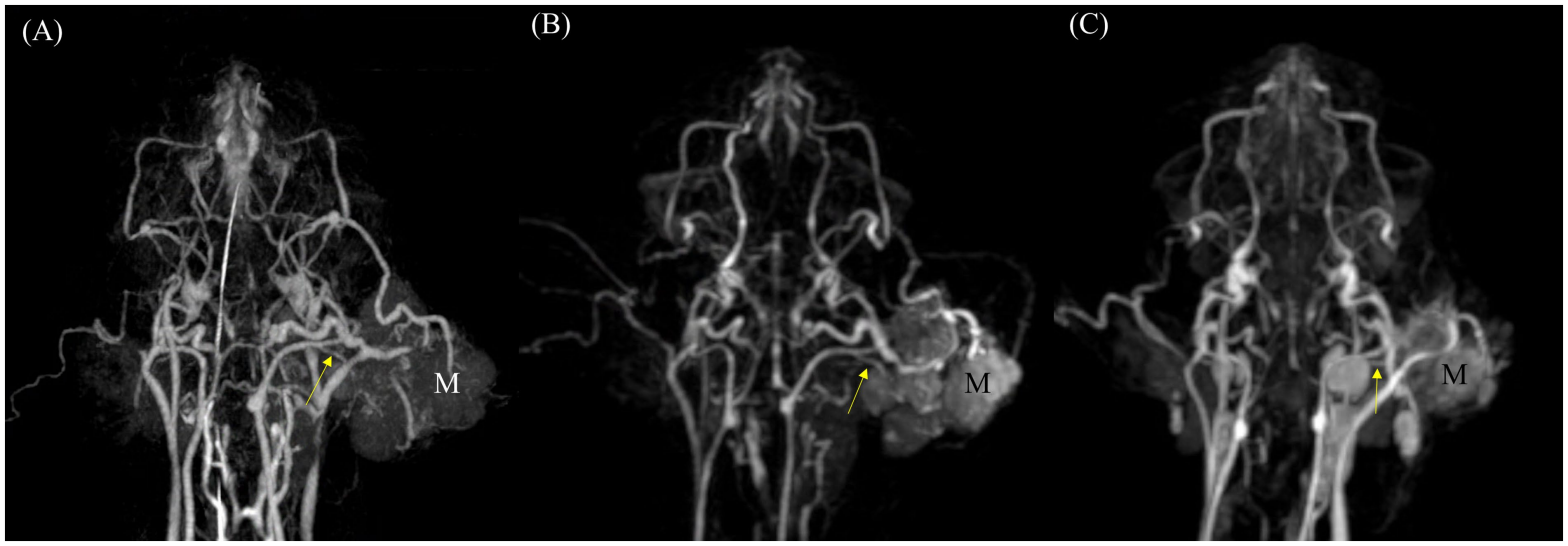
Comparison of feeding vessels in a ceruminous adenocarcinoma originating from the left ear in a cat using dorsal maximum intensity projection (MIP) of CT angiography (CTA) and time-resolved imaging of contrast kinetics (TRICKS). **(A)** Arterial-phase CTA and **(B)** dorsal MIP image from TRICKS show the left caudal auricular artery (yellow arrow), which appears to be the feeding vessel supplying the tumor (M). **(C)** Follow-up imaging 1 month after radiation therapy reveals a significantly reduced tumor size, along with altered trajectories of the peritumoral vessels compared to the initial examination with caudal auricular artery (yellow arrow).

**Figure 7:**
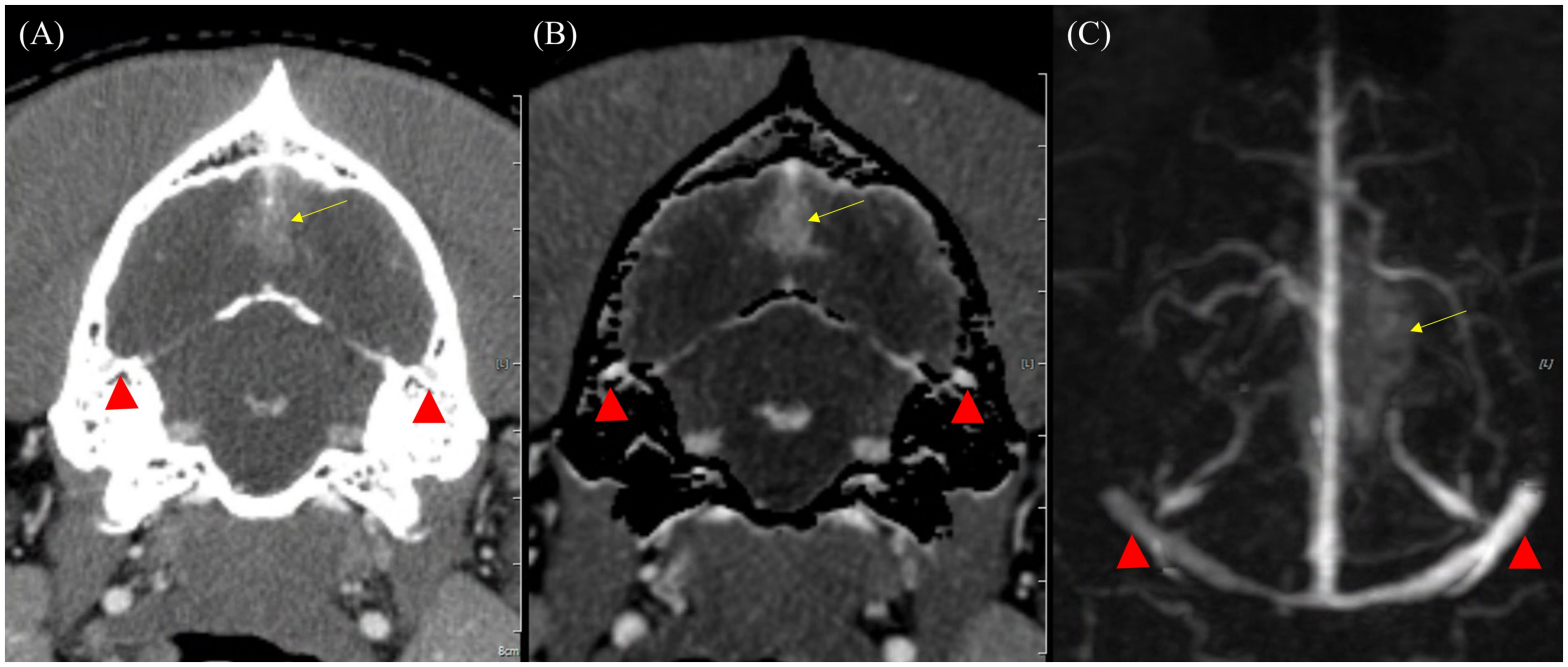
Comparison of bone-related artifacts in venous phase of CT angiography (CTA) and Time-resolved imaging of contrast kinetics (TRICKS) in a dog with a suspected meningioma. **(A)** Transverse plane of venous phase CT image showing the bilateral temporal sinuses (red arrowheads) obscured by the adjacent temporal bones, resulting in limited vascular visibility. **(B)** Bone-subtracted CTA image demonstrating partial removal of bony structures. However, incomplete subtraction results in residual artifacts, resulting in indistinct vascular boundaries (red arrowheads). **(C)** Dorsal maximum intensity projection of TRICKS image of the same region, demonstrating well-defined and homogeneous high-signal bilateral temporal sinuses, unaffected by bone-related artifacts (red arrowheads). In addition, the bilateral transverse sinuses, sigmoid sinuses, cerebral sagittal sinus, and dorsal cerebral vein are also clearly visualized. A suspected meningioma is observed in the dorsal occipital lobe (yellow arrow).

**Figure 8:**
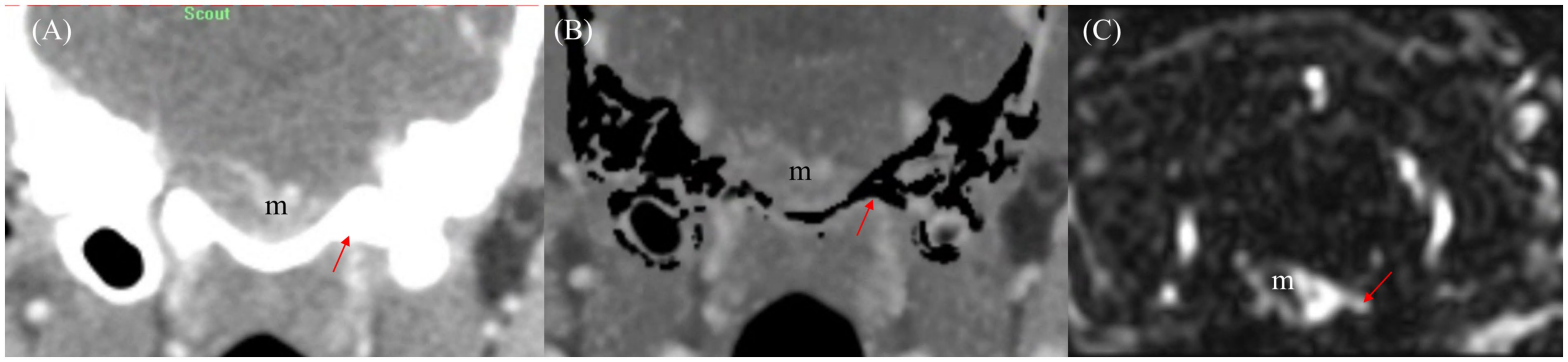
Comparison of draining vessel visualization using transverse planes of CT angiography (CTA) and Time-resolved imaging of contrast kinetics (TRICKS) in a dog with basal meningioma (m) at the brainstem level. **(A)** Venous phase CT image showing difficulty in assessing the venous drainage due to bone-related interference. The draining vein (red arrow) is partially obscured by surrounding bony structures. **(B)** Bone-subtracted CTA image demonstrating improved visibility of the draining vein (red arrow) by reducing bony interference. However, partial removal of osseous structures results in residual subtraction artifacts. **(C)** TRICKS image providing clearly delineating of the connection between the meningioma (m) and the draining vein (red arrow) without bone-related artifact, allowing for enhanced vascular assessment.

The qualitative evaluation of tumor-associated vessels conducted for tumor-vessel differentiation, intratumoral blood flow visualization, conspicuity of the vessel from bone, and feeding and draining vessel identification are presented in Table 7. The feeding and draining vessels varied depending on the tumor involvement in each patient. However, the most commonly observed feeding arteries included the caudal auricular artery, lateral nasal artery, rostral cerebral artery, descending palatine artery, basilar artery, maxillary artery, and inferior alveolar artery. The identified draining vessels comprised the maxillary vein, lateral nasal vein, dorsal cerebral vein, dorsal sagittal sinus, straight sinus, dorsal external ophthalmic vein, dorsal nasal vein, frontal cerebral diploic vein, vertebral vein, and facial vein, which were assessed in the evaluation. There was no significant difference between CTA and TRICKS in tumor-vessel differentiation, intratumoral blood flow visualization, or feeding and draining vessel identification (Figure 6), indicating that both imaging modalities provided comparable visualization of these vascular characteristics. However, TRICKS showed a significant greater conspicuity of blood vessels from the adjacent bone structures compared to CTA, suggesting that it can provide improved visualization of vascular structures in regions where bony interference affects vascular assessment (Figures 7, 8).

**Table 7:** Qualitative evaluation of tumor-associated feeding and draining vessels in CT angiography (CTA) and time-resolved imaging of contrast kinetics (TRICKS).

Evaluation criteria	CTA		TRICKS		Z-score	*p*-value
	Mean ± SD	Median (min, max)	Mean ± SD	Median (min, max)		
Tumor-vessel differentiation	2.8 ± 0.42	3.0 (2,3)	2.9 ± 0.32	3.0 (2,3)	−1.000	0.317
Intratumoral blood flow visualization	2.4 ± 0.70	2.5 (1,3)	2.4 ± 0.84	3.0 (1,3)	0.000	1.000
Conspicuity the vessel from bone	2.1 ± 0.74	2.0 (1,3)	3.0 ± 0.00	3.0 (3,3)	−2.460	0.014*
Feeding and draining vessel identification	2.9 ± 0.32	3.0 (2,3)	3.0 ± 0.00	3.0 (3,3)	−1.000	0.317

*p-value < 0.05. SD, standard deviation; min, minimal value; max, maximal value.

## 4 Discussion

In this study, vascular imaging using CTA and TRICKS were compared in patients with head and neck tumors to assess intracranial and extracranial vessels. Although CTA provided excellent visualization and delineation with noise-free image quality within a significantly short acquisition time, TRICKS showed superior performance in evaluating the structure of tumor- associated vessels while achieving a diagnostic performance comparable to CTA. Additionally, TRICKS offered the advantage of reduced bone-related artifacts, enhancing the visualization of vascular structures in complex anatomical regions. To the best of our knowledge, this is the first study to apply TRICKS for the assessment of tumor-associated vasculature in dogs with naturally occurring neoplasms. While previous studies have investigated TRICKS in normal dogs, its application in tumor-bearing patients had not been explored. Our findings suggest that TRICKS could serve as a valuable imaging modality for evaluating vascular characteristics in veterinary oncology, particularly for surgical and radiation therapy planning.

Signal intensity is important in vascular imaging, as it directly affects the clarity of vessel visualization. To evaluate this, the SI of the basilar artery and transverse sinus—representative intracranial arteries and veins— were compared between CTA and TRICKS. While the overall SI between the two modalities showed no significant difference, CTA showed significantly higher SI in the basilar artery compared to TRICKS. This difference may be attributed to the intrinsic characteristics of CTA, which enables the acquisition of images at the peak contrast enhancement phase through a rapid scan. Additionally, the fast injection rate and high concentration of contrast agent in CTA can lead to distinct contrast intensity differences between the arterial and venous phases (26). In contrast, TRICKS captures a continuous series of dynamic phases after contrast injection, allowing for uniform contrast enhancement across different vascular structures. This continuous acquisition reduces the impact of timing variability, providing a more consistent depiction of arterial and venous structures. Consequently, while CTA may offer superior arterial phase contrast due to its peak enhancement capture, TRICKS provides a more stable and reliable visualization of both arterial and venous phases, minimizing the influence of variable scan timing.

This result was further supported by the qualitative evaluation, where vascular visibility, delineation, connectivity, artifact associated with bone, and overall image quality were assessed through both the combined overall vascular structures and the vessel-by-vessel comparisons. Consistent with the signal intensity findings, CTA demonstrated significantly superior diagnostic performance in terms of vascular visibility, particularly for arterial structures. Specifically, CTA showed significantly better visibility in 5 out of 7 intracranial arteries, 5 out of 5 extracranial arteries, 2 out of 6 intracranial veins, and 1 out of 3 extracranial veins. These results align with the higher SI observed in the basilar artery on CTA, reinforcing its advantage in enhancing arterial visualization due to peak contrast timing and rapid acquisition.

Meanwhile, in venous visualization, TRICKS demonstrated comparable or superior visibility to CTA. This result can be explained by differences in acquisition timing between the two modalities. CTA relies on a single-phase acquisition, and in this study, the venous phase was acquired using a test bolus technique rather than bolus tracking. This method allowed for a more precise determination of the optimal timing for venous phase imaging, minimizing timing variability. However, since CTA still captures only a single phase, it remains susceptible to contrast circulation variations that may affect venous enhancement. In contrast, TRICKS acquires a continuous series of time-resolved phases for a longer duration, up to approximately 2 min after contrast injection. This method ensures capturing the dynamic progression of contrast enhancement and consistent venous visualization across different time points and reducing the risk of missing peak venous enhancement (27, 28).

The differences in visibility between CTA and TRICKS were further reflected in the evaluation of vascular delineation. When assessed for the entire vascular system, CTA received higher overall scores. In vessel-by-vessel comparisons, CTA demonstrated superior delineation in 4 out of 7 intracranial arteries and 4 out of 5 extracranial arteries. However, no veins showed higher scores for CTA compared to TRICKS. In contrast, TRICKS exhibited better delineation in specific vessels, with all intracranial veins receiving higher scores compared to CTA. This finding is associated with the strength of TRICKS, which achieved a higher score in the evaluation of artifact-related interference from bone compared to CTA.

Image quality is determined by factors such as contrast resolution, spatial resolution, noise levels, and the presence of artifacts. CTA can typically achieve a higher signal to noise ratio due to its higher photon flux and maintain low noise, when higher radiation dose is used (29). Additionally, CTA typically provides good image quality due to the inherently high spatial resolution and optimized contrast enhancement. However, image quality in CTA can be affected by beam-hardening artifacts generated from the contrast agent, particularly in high density structures, such as intracranial CTA, where densely packed bone and vessels increase artifact susceptibility.

Many intracranial venous sinuses are located within or adjacent to the dorsal part of the occipital bone, where the bony structures can interfere with vessel visualization in CTA. The transverse sinus, for instance, runs within the transverse canal of the occipital bone, while the dorsal sagittal sinus connects to the transverse sinus via the foramen sinus sagittalis dorsalis. Similarly, the temporal sinus lies between the petrous and squamous parts of the temporal bone, and the sigmoid sinus travels in an arc-like path around the caudal end of the petrous portion of the temporal bone (30). Since intracranial veins are either enclosed within bony canals or closely associated with surrounding bone, the differentiation between bone and contrast-enhanced blood vessels in CTA can be challenging due to their similarly high HU values. This also affects bone subtraction techniques, which may not only remove bone structures but also inadvertently eliminate adjacent vessels, thereby reducing vascular delineation. Consequently, despite its advantages in spatial resolution and signal to noise ratio, CTA is limited in visualizing intracranial veins located near dense bone interfered with vascular delineation due to beam-hardening artifacts and challenges in bone subtraction. This limitation was particularly evident in, where surrounding bony structures.

Meanwhile, TRICKS reduced bone-related artifacts, allowing for improved visualization of venous structures. Therefore, TRICKS recorded significantly higher scores in artifact reduction across all vessels, particularly in the six intracranial veins, where it showed a significant advantage. Interestingly, these veins correspond to those where TRICKS received higher scores in delineation, likely due to their anatomical locations and the reduced interference from adjacent bone. However, despite this advantage, TRICKS exhibited more noticeable noise and generally has higher noise levels compared to CTA. Advancements in MRI technology, such as higher Tesla scanners and extended acquisition times, may help minimize noise and improve image quality.

The overall vascular connectivity showed no significant difference between CTA and TRICKS when evaluated across all vessels, as both modalities provided continuous visualization of the vasculature without noticeable interruptions. This can be attributed to several technical factors in each modality. CTA utilizes thin slice thickness during image acquisition, allowing for high-resolution visualization and smooth MIP reconstruction of vessels, while TRICKS employs a 3D dynamic imaging technique, capturing volumetric data in multiple time phases. However, when assessed individually, TRICKS was significantly superior connectivity in 4 out of 6 intracranial veins. The time-resolved nature of TRICKS contributed to maintaining vessel connectivity, particularly in smaller veins with complex multidirectional flow patterns. In summary, the transverse sinus and sigmoid sinus can be well visualized in both CTA and TRICKS, while TRICKS provided enhanced connectivity in other intracranial venous structures.

In tumor assessment, identifying feeding and draining vessels, distinguishing tumor vasculature, and visualizing intratumoral blood flow are crucial for surgical and treatment planning. As a result, CTA is often needed in addition to MRI to provide detailed vascular mapping. However, in veterinary patients, additional CTA requires prolonged anesthesia and patient repositioning, leading to increased procedural time. In this study, TRICKS showed no significant difference compared to CTA in visualizing the tumor associated vessels in tumor patients. This suggests that adding the TRICKS sequence to a standard MRI scan may eliminate the need for additional CTA examinations when assessing peritumoral vasculature. Additionally, TRICKS demonstrated significantly higher accuracy than CTA in differentiating blood vessels from bone, potentially overcoming the limitations of CTA in vascular imaging.

The clinical usefulness of TRICKS has not reported in veterinary medicine; therefore, these findings could not be compared to other veterinary studies. However, studies in human patients have reported the advantages of MR angiography over CTA (9, 31–35). These studies have also explored the use of time-resolved MRA in identifying feeding and draining vessels, not only in tumors but also in vascular malformations. Among these studies, one comparing MR angiography of tumor-related vasculature with CTA reported that time-resolved MRA is a reliable imaging modality for tumor evaluation, showing comparable performance to CTA in identifying feeding arteries and arteriovenous fistulas (AVFs) (35). Unlike CTA, which provides single-phase imaging, TRICKS includes multiple arterial and venous phases, allowing for dynamic analysis. Additionally, while CTA showed slightly higher overall image quality than MRA, time-resolved MRA visualized a greater number of feeding arteries and AVFs than CTA in certain human patients. These findings support the potential utility of TRICKS as a complementary imaging modality for vascular evaluation in tumor patients.

In addition, when clinical and microenvironmental perspectives of tumor vasculature were evaluated alongside digital photography and high-resolution intravital microscopy, MRA enabled noninvasive quantification of tumor angiogenesis (9). Since angiogenesis plays a crucial role in tumor growth, TRICKS could have potential in assessing the microenvironment of tumor vasculature. Furthermore, when combined with dynamic contrast-enhanced MR imaging, TRICKS could improve the understanding of tumor blood supply dynamics and treatment response, facilitating more accurate treatment planning.

This study revealed morphological differences in the basilar artery between dogs and cats. In the nine dogs, the basilar artery varied between a tortuous and straight configuration, whereas it was consistently straight in the cat. Although based on a single cat, this finding is comparable to previous studies, including one involving four cats, which reported a greater tendency for tortuosity in dogs (36). A larger study of 50 cats further identified a significant sex-based variation, with males predominantly exhibiting a straight configuration and females more frequently showing a tortuous shape (37). This anatomical difference in the basilar artery between dogs and cats can be related with blood supply to the brain: While cats receive contributions solely from the bilateral vertebral arteries, dogs may also receive input from the occipital artery. This pattern was confirmed in the dogs included in our study, where both the occipital artery and vertebral arteries contributed to the basilar artery. In contrast, in the cat, only the vertebral arteries were involved.

Regarding CTA evaluation, the only subject whose basilar artery received a score of 2 or lower in all categories (visibility, delineation, and connectivity) was the cat. This result is likely due to the anatomical proximity of the basilar artery to the basal part of the occipital bone in cats, which led to its removal during post-processing when bone subtraction was applied. Further research with a larger sample size of cats is needed to determine whether this phenomenon is consistently observed across feline subjects.

There are several limitations in this study. First, only a small number of 10 subjects are included in this study, with only a single case of feline patient. This small sample size made it difficult to generalize imaging characteristics in cats. Future studies focusing exclusively on feline subjects may be necessary to better understand their vascular imaging patterns. Both CTA and TRICKS may have produced inconsistent results due to variations in imaging protocols and parameters. In particular, CTA is highly dependent on the timing of contrast injection, which may vary slightly depending on the operator, potentially leading to significant differences in image quality and interpretation scores. This limitation is inherent in retrospective studies involving real patients. Therefore, future studies should be conducted using a standardized protocol with uniform imaging parameters to improve consistency. Although TRICKS demonstrated notable diagnostic advantages, it has inherent limitations compared to CTA, particularly in spatial resolution. The slice thickness of TRICKS is approximately 2 mm, which is significantly thicker than the 0.5 mm slices of CTA. This results in reduced differentiation in the dorsal-ventral direction. However, with higher-specification MRI scanners (3 T or above), the combination of reduced acquisition time and improved resolution may allow TRICKS to serve as a complementary imaging modality alongside CTA. Lastly, while feeding and draining vessels were assessed using CTA, no cases underwent post-mortem examination. This limitation prevents a definitive evaluation of the actual vascular architecture and its correlation with imaging findings. Future studies incorporating histopathological validation could provide a more comprehensive understanding of tumor-associated vasculature.

## 5 Conclusion

This study evaluated the diagnostic utility of TRICKS compared to CTA for assessing intracranial and extracranial vascular structures in veterinary patients. TRICKS provided comparable visualization of vascular structures, particularly in venous anatomy, while also offering dynamic imaging capabilities that allow for a more comprehensive assessment of blood flow over time. TRICKS effectively visualized both feeding and draining vessels associated with tumors, showing performance similar to CTA in identifying vascular structures related to tumor circulation. Additionally, TRICKS exhibited a distinct advantage in differentiating vessels from surrounding bone structures, reducing the interference of bone-related artifacts that can affect vessel delineation in CTA. While CTA provided superior spatial resolution and clearer visualization of arterial structures, TRICKS enabled a more continuous assessment of contrast dynamics, minimizing the impact of timing variations seen in CTA. These findings suggest that TRICKS can serve as a valuable imaging modality for vascular assessment in veterinary neuro-oncology, particularly for evaluating venous structures and tumor-related vasculature.

## Data Availability

The raw data supporting the conclusions of this article will be made available by the authors, without undue reservation.

## Supplementary material

SUPPLEMENTARY TABLE S1 Qualitative evaluation of intracranial and extracranial vascular structures in individual patients using computed tomography angiography and time-resolved imaging of contrast kinetics magnetic resonance angiography.

## References

[1] Song RB Vite CH Bradley CW Cross JR. Postmortem evaluation of 435 cases of intracranial neoplasia in dogs and relationship of neoplasm with breed, age, and body weight. J Vet Intern Med. (2013) 27:1143–52. doi: 10.1111/jvim.12136, PMID: 23865437

[2] Zaki FA Hurvitz AI. Spontaneous neoplasms of the central nervous system of the cat. J Small Anim Pract. (1976) 17:773–82. doi: 10.1111/j.1748-5827.1976.tb06943.x, PMID: 1034854

[3] Mariani C. Tumours of the nervous system In: Dobson J Lascelles D, editors. Manual of canine and feline oncology. third ed. UK: BSAVA (2011). 329–40.

[4] Rossmeisl JH Jr Pancotto TE. Tumors of the nervous system In: Vail DM Thamm DH Liptak JM, editors. Withrow & MacEwen’s small animal clinical oncology. sixth ed. Missouri: Elsevier (2020). 657.

[5] Choudhary OP Infant SS As V Chopra H Manuta N. Exploring the potential and limitations of artificial intelligence in animal anatomy. Ann Anat. (2025) 258:152366. doi: 10.1016/j.aanat.2024.152366, PMID: 39631569

[6] Vickram AS Infant SS Priyanka CH. AI-powered techniques in anatomical imaging: impacts on veterinary diagnostics and surgery. Ann Anat. (2025) 258:152355. doi: 10.1016/j.aanat.2024.152355, PMID: 39577814

[7] Hu H Barker A Harcourt-Brown T Jeffery. Systematic review of brain tumor treatment in dogs. J Vet Intern Med. (2015) 29:1456–63. doi: 10.1111/jvim.13617, PMID: 26375164 PMC4895648

[8] Barker R Fareedi S Thompson D Saunders D. The use of CT angiography in the preoperative planning. Childs Nerv Syst. (2009) 25:955–9. doi: 10.1007/s00381-009-0904-9, PMID: 19484250

[9] Vliet MV Dijike CF Wielopolski PA Hagen TL Veenland JF Preda A. MR angiography of tumor-related vasculature: from the clinic to the micro-environment. Radiographics. (2005) 25:S85–97. doi: 10.1148/rg.25si055512, PMID: 16227499

[10] Shaban S Huasen B Haridas A Killingsworth M Worthington J Jabbour P. Digital subtraction angiography in cerebrovascular disease: current practice and perspectives on diagnosis, acute treatment and prognosis. Acta Neurol Belg. (2022) 122:763–80. doi: 10.1007/s13760-021-01805-z, PMID: 34553337

[11] Lufft V Hoogestraat-Lufft L Fels LM Egbeyong-Baiyee D Tusch G Galanski M. Contrast media nephropathy: intravenous CT angiography versus intraarterial digital subtraction angiography in renal artery stenosis: a prospective randomized trial. Am J Kidney Dis. (2002) 40:236–42. doi: 10.1053/ajkd.2002.34501, PMID: 12148095

[12] Goic JB Koenigshof AM McGuire LD Klinger AC Beal MW. A retrospective evaluation of contrast-induced kidney injury in dogs (2006–2012). J Vet Emerg Crit Care (San Antonio). (2016) 26:713–9. doi: 10.1111/vec.12511, PMID: 27557489

[13] Wilms G Bosmans H Marchal G Demaerel P Goffin J Plets C. Magnetic resonance angiography of supratentorial tumours: comparison with selective digital subtraction angiography. Neuroradiology. (1995) 37:42–7. doi: 10.1007/BF00588518, PMID: 7708188

[14] Kane GC Stanson AW Kalnicka D Rosenthal DW Lee CU Textor SC. Comparison between gadolinium and iodine contrast for percutaneous intervention in atherosclerotic renal artery stenosis: clinical outcomes. Nephrol Dial Transplant. (2008) 23:1233–40. doi: 10.1093/ndt/gfm725, PMID: 18256017

[15] Dickinson PJ. Advances in diagnostic and treatment modalities for intracranial tumors. J Vet Intern Med. (2014) 28:1165–85. doi: 10.1111/jvim.12370, PMID: 24814688 PMC4857954

[16] Duffis EJ Gandhi CD Prestigiacomo CJ Abruzzo T Albuquerque F Bulsara KR. Head, neck, and brain tumor embolization guidelines. J Neurointerv Surg. (2012) 4:251–5. doi: 10.1136/neurintsurg-2012-010350, PMID: 22539531 PMC3370378

[17] Hong S Kim S Namgoong M Kwon M Yoon J Choi J. Magnetic resonance angiography for cerebral artery and venous visualization in dogs: a comparative study of time-of-flight, phase-contrast, and time-resolved imaging of contrast kinetics sequences. Front Vet Sci. (2025) 12:1533130. doi: 10.3389/fvets.2025.1533130, PMID: 40041665 PMC11876181

[18] Tomandl BF Hammen T Klotz E Ditt H Stemper B Lell M. Bone-subtraction CT angiography for the evaluation of intracranial aneurysms. Am J Neuroradiol. (2006) 27:55–9. PMID: 16418356 PMC7976055

[19] Lell M Anders K Klotz E Ditt H Bautz W Tomandl BF. Clinical evaluation of bone-subtraction CT angiography (BSCTA) in head and neck imaging. Eur Radiol. (2006) 16:889–97. doi: 10.1007/s0330-005-0032-1, PMID: 16267665

[20] Venema HW Hulsmans FJ Heeten GJ. CT angiography of the circle of Willis and Intracranial internal carotid arteries: maximum intensity projection with matched mask bone elimination— feasibility study. Radiology. (2001) 218:893–8. doi: 10.1148/radiology.218.3.r01mr30893, PMID: 11230672

[21] An S Hwang G Kim R Cha J Lee HC Hwang TS. Optimizing contrast protocol for bone-subtraction CT angiography of intracranial arteries in normal dogs using 160-slice CT. Vet Med Sci. (2023) 9:2504–12. doi: 10.1002/vms3.1252, PMID: 37766491 PMC10650334

[22] Lee M Ko M Ahn J Ahn J Yu J Chang J. Evaluation of the abdominal aorta and external iliac arteries using three-dimensional time-of-flight, three dimensional electrocardiograph-gated fast spin-Echo, and contrast-enhanced magnetic resonance angiography in clinically healthy cats. Front Vet Sci. (2022) 9:819627. doi: 10.3389/fvets.2022.819627, PMID: 35782562 PMC9249124

[23] Zhang N Fan Z Luo N Bi X Zhao Y An J. Noncontrast MR angiography (MRA) of Infragenual arteries using flow-sensitive dephasing (FSD)-prepared steady-state free precession (SSFP) at 3.0T: comparison with contrast-enhanced MRA. J Magn Reson Imaging. (2016) 43:364–72. doi: 10.1002/jmri.25003, PMID: 26185106 PMC4715799

[24] Asbach P Meade MD Sattenberg RJ Kelssen C Huppertz A Heidenreich JO. Continuously moving table aorto-iliofemoral run-off contrast-enhanced magnetic resonance angiography: image quality analysis in comparison to the multistep acquisition. Acta Radiol. (2014) 55:266–72. doi: 10.1177/0284185113498535, PMID: 24078458

[25] Shoukri MM. Measures of interobserver agreement and reliability. Boca Raton, Florida: Chapman & Hall/CRC (2011).

[26] Shirasaka T Hiwatashi A Yamashita K Kondo M Hamasaki H Shimomiya Y. Optimal scan timing for artery–vein separation at whole-brain CT angiography using a 320-row MDCT volume scanner. Br J Radiol. (2017) 90:20160634. doi: 10.1259/bjr.20160634, PMID: 27995807 PMC5685121

[27] Laub G. Principles of contrast enhanced mr angiography. Basic and clinical applications. Magn Reson Imaging Clin N Am. (1999) 7:783–95. doi: 10.1016/S1064-9689(21)00521-3 10631678

[28] Doltra A Skorin A Hamdan A Schnackenburg B Gebker R Klein C. Comparison of gadolinium dose and acquisition time for late gadolinium enhancement at 3.0 T. Eur Radiol. (2014) 24:2192–200. doi: 10.1007/s00330-014-3213-y, PMID: 24828537

[29] Murdef HM Jumhur AS Hamim MH Rashah MA Alzanati MM Almuhameth NM. Comparison of CT and MRI for brain imaging: review article. J Int Crisis Risk Commun Res. (2024) 7:338–51. doi: 10.63278/jicrcr.vi.364

[30] Hermanson JW Lahunta A Evans HE. The skeleton. Miller’s anatomy of the dog. fifth ed. Missouri: Elsevier (2020). 182 p.

[31] Bista B Yousra A Bosemani T Gedeon D Bista A Shrestha S. The utility of time resolved magnetic resonance angiography in differentiating vascular malformations. Clin Imaging. (2023) 101:150–5. doi: 10.1016/j.clinimag.2023.06.010, PMID: 37364365

[32] Romano A Tavanti F Espagnet MC Terenzi V Cassoni A Suma G. The role of time-resolved imaging of contrast kinetics (TRICKS) magnetic resonance angiography (MRA) in the evaluation of head–neck vascular anomalies; a preliminary experience. Dentomaxillofac Radiol. (2015) 44:20140302. doi: 10.1259/dmfr.20140302, PMID: 25410709 PMC4614168

[33] Hassanien OA Ghieda UE Younes RL Shaban EA. Facial vascular anomalies; MRI and TRICKS-MR angiography diagnostic approach. Egypt J Radiol Nucl Med. (2017) 48:885–95. doi: 10.1016/j.ejrnm.2017.08.013, PMID: 40292123

[34] Razek AA Gaballa G Megahed AS Elmogy E. Time resolved imaging of contrast kinetics (TRICKS) MR angiography of arteriovenous malformations of head and neck. Eur J Radiol. (2013) 82:1885–91. doi: 10.1016/j.ejrad.2013.07.007, PMID: 23928233

[35] Wu G Jin T Li T Morelli J Li X. High spatial resolution time-resolved magnetic resonance angiography of lower extremity tumors at 3T. Medicine (Baltimore). (2016) 95:e4894. doi: 10.1097/MD.0000000000004894, PMID: 27631262 PMC5402605

[36] Gillian LA. Extra- and intra-cranial blood supply to brains of dog and cat. Am J Anat. (1976) 146:237–53. doi: 10.1002/aja.1001460303, PMID: 941852

[37] Salvador-Gomes M Hernandez JM Alonso L Figueiredo MA. Morphology and main branches of the basilar artery in cats. Rev Bras Med Vet. (2012) 34:206–12. Available at: https://bjvm.org.br/BJVM/article/view/727

