# Supplementary material for: Time-resolved imaging of contrast kinetics magnetic resonance angiography for the assessment of vascular characteristics in intracranial and extracranial tumors in veterinary patients
Source: Front Vet Sci. 2025 May 21;12:1579582. doi: 10.3389/fvets.2025.1579582 (PMC12135079; doi:10.3389/fvets.2025.1579582)
Supplement: SUPPLEMENTARY TABLE S1 — Qualitative evaluation of intracranial and extracranial vascular structures in individual patients using computed tomography angiography and time-resolved imaging of contrast kinetics magnetic resonance angiography. [file Table_1.docx]

Qualitative Evaluation (score 1-4)

| Patient | 1 |  |  |  |  |  |  |
| --- | --- | --- | --- | --- | --- | --- | --- |
|  | Vessels | Modality | Vascular visibility | Vascular delineation | Vascular connectivity | Vascular artifact associated adj. bone | Image quality |
|  | Basilar artery | CTA | 2 | 2 | 2 | 1 | 4 |
|  |  | TRICKS | 2 | 2 | 2 | 4 | 3 |
|  | Rostral cerebral artery | CTA | 3 | 4 | 4 | 4 | 4 |
|  |  | TRICKS | 2 | 2 | 3 | 4 | 3 |
|  | Middle cerebral artery | CTA | 3 | 4 | 4 | 4 | 4 |
|  |  | TRICKS | 1 | 1 | 1 | 4 | 3 |
|  | Caudal cerebral artery | CTA | 3 | 2 | 3 | 4 | 4 |
|  |  | TRICKS | 3 | 2 | 3 | 4 | 3 |
|  | Rostral cerebellar artery | CTA | 3 | 2 | 3 | 4 | 4 |
|  |  | TRICKS | 3 | 2 | 4 | 4 | 3 |
|  | Caudal communicating artery | CTA | 3 | 2 | 3 | 4 | 4 |
|  |  | TRICKS | 3 | 2 | 4 | 4 | 3 |
|  | Internal carotid artery | CTA | 1 | 1 | 1 | 4 | 4 |
|  |  | TRICKS | 1 | 1 | 1 | 4 | 3 |
|  | Dorsal sagittal sinus | CTA | 2 | 1 | 1 | 1 | 4 |
|  |  | TRICKS | 3 | 3 | 4 | 4 | 3 |
|  | Transverse sinus | CTA | 2 | 2 | 1 | 1 | 4 |
|  |  | TRICKS | 3 | 3 | 3 | 4 | 3 |
|  | Temporal sinus | CTA | 1 | 1 | 1 | 1 | 4 |
|  |  | TRICKS | 3 | 3 | 3 | 4 | 3 |
|  | Sigmoid sinus | CTA | 1 | 1 | 1 | 1 | 4 |
|  |  | TRICKS | 3 | 3 | 3 | 4 | 3 |
|  | Straight sinus | CTA | 1 | 1 | 1 | 1 | 4 |
|  |  | TRICKS | 3 | 3 | 3 | 4 | 3 |
|  | Dorsal cerebral vein | CTA | 2 | 2 | 2 | 1 | 4 |
|  |  | TRICKS | 4 | 4 | 3 | 4 | 3 |
|  | Infraorbital artery | CTA | 4 | 4 | 4 | 4 | 4 |
|  |  | TRICKS | 3 | 3 | 3 | 4 | 3 |
|  | Descending palatine artery | CTA | 4 | 4 | 4 | 4 | 4 |
|  |  | TRICKS | 3 | 3 | 4 | 4 | 3 |
|  | Maxillary artery | CTA | 4 | 4 | 4 | 4 | 4 |
|  |  | TRICKS | 3 | 3 | 4 | 4 | 3 |
|  | Caudal deep temporal artery | CTA | 1 | 1 | 1 | 1 | 1 |
|  |  | TRICKS | 2 | 2 | 2 | 4 | 2 |
|  | Caudal auricular artery | CTA | 4 | 4 | 4 | 4 | 4 |
|  |  | TRICKS | 4 | 4 | 3 | 4 | 3 |
|  | Maxillary vein | CTA | 4 | 4 | 4 | 4 | 4 |
|  |  | TRICKS | 4 | 4 | 4 | 4 | 3 |
|  | Superficial temporal vein | CTA | 4 | 3 | 4 | 4 | 4 |
|  |  | TRICKS | 3 | 3 | 3 | 4 | 3 |
|  | Dorsal external ophthalmic vein | CTA | 4 | 4 | 4 | 4 | 4 |
|  |  | TRICKS | 4 | 4 | 4 | 4 | 3 |
| Patient | 2 |  |  |  |  |  |  |
|  |  |  |  |  |  |  |  |
|  |  |  | Vascular visibility | Vascular delineation | Vascular connectivity | Vascular artifact associated adj. bone | Image quality |
|  | Basilar artery | CTA | 4 | 4 | 4 | 4 | 4 |
|  |  | TRICKS | 4 | 3 | 4 | 4 | 3 |
|  | Rostral cerebral artery | CTA | 4 | 4 | 4 | 4 | 4 |
|  |  | TRICKS | 4 | 4 | 4 | 4 | 3 |
|  | Middle cerebral artery | CTA | 4 | 4 | 4 | 4 | 4 |
|  |  | TRICKS | 3 | 3 | 3 | 4 | 3 |
|  | Caudal cerebral artery | CTA | 4 | 4 | 4 | 4 | 3 |
|  |  | TRICKS | 3 | 3 | 3 | 4 | 3 |
|  | Rostral cerebellar artery | CTA | 4 | 4 | 4 | 4 | 4 |
|  |  | TRICKS | 3 | 3 | 3 | 4 | 3 |
|  | Caudal communicating artery | CTA | 4 | 3 | 4 | 4 | 4 |
|  |  | TRICKS | 3 | 3 | 3 | 4 | 3 |
|  | Internal carotid artery | CTA | 4 | 4 | 4 | 4 | 4 |
|  |  | TRICKS | 4 | 3 | 4 | 4 | 3 |
|  | Dorsal sagittal sinus | CTA | 4 | 3 | 3 | 2 | 3 |
|  |  | TRICKS | 4 | 4 | 4 | 4 | 3 |
|  | Transverse sinus | CTA | 2 | 2 | 2 | 2 | 2 |
|  |  | TRICKS | 4 | 3 | 4 | 4 | 3 |
|  | Temporal sinus | CTA | 2 | 2 | 2 | 2 | 2 |
|  |  | TRICKS | 4 | 4 | 4 | 4 | 3 |
|  | Sigmoid sinus | CTA | 2 | 2 | 2 | 2 | 3 |
|  |  | TRICKS | 4 | 4 | 4 | 4 | 3 |
|  | Straight sinus | CTA | 2 | 2 | 3 | 2 | 3 |
|  |  | TRICKS | 4 | 3 | 4 | 4 | 3 |
|  | Dorsal cerebral vein | CTA | 2 | 2 | 3 | 2 | 3 |
|  |  | TRICKS | 4 | 3 | 3 | 4 | 3 |
|  | Infraorbital artery | CTA | 4 | 4 | 4 | 4 | 4 |
|  |  | TRICKS | 4 | 4 | 4 | 4 | 3 |
|  | Descending palatine artery | CTA | 4 | 3 | 4 | 3 | 4 |
|  |  | TRICKS | 4 | 3 | 4 | 4 | 3 |
|  | Maxillary artery | CTA | 4 | 4 | 4 | 4 | 4 |
|  |  | TRICKS | 4 | 4 | 4 | 4 | 3 |
|  | Caudal deep temporal artery | CTA | 4 | 4 | 4 | 4 | 4 |
|  |  | TRICKS | 3 | 4 | 4 | 4 | 3 |
|  | Caudal auricular artery | CTA | 4 | 4 | 4 | 4 | 4 |
|  |  | TRICKS | 3 | 3 | 4 | 4 | 3 |
|  | Maxillary vein | CTA | 4 | 4 | 4 | 4 | 4 |
|  |  | TRICKS | 4 | 4 | 4 | 4 | 3 |
|  | Superficial temporal vein | CTA | 4 | 3 | 3 | 3 | 4 |
|  |  | TRICKS | 3 | 3 | 4 | 4 | 3 |
|  | Dorsal external ophthalmic vein | CTA | 4 | 4 | 4 | 4 | 4 |
|  |  | TRICKS | 4 | 4 | 4 | 4 | 3 |
|  |  |  |  |  |  |  |  |
| Patient | 3 |  |  |  |  |  |  |
|  |  |  |  |  |  |  |  |
|  |  |  | Vascular visibility | Vascular delineation | Vascular connectivity | Vascular artifact associated adj. bone | Image quality |
|  | Basilar artery | CTA | 4 | 4 | 4 | 4 | 4 |
|  |  | TRICKS | 4 | 4 | 4 | 4 | 3 |
|  | Rostral cerebral artery | CTA | 4 | 4 | 4 | 4 | 4 |
|  |  | TRICKS | 4 | 4 | 4 | 4 | 3 |
|  | Middle cerebral artery | CTA | 4 | 4 | 4 | 4 | 4 |
|  |  | TRICKS | 4 | 4 | 4 | 4 | 3 |
|  | Caudal cerebral artery | CTA | 4 | 4 | 4 | 4 | 4 |
|  |  | TRICKS | 2 | 2 | 3 | 4 | 3 |
|  | Rostral cerebellar artery | CTA | 4 | 4 | 4 | 4 | 4 |
|  |  | TRICKS | 4 | 3 | 4 | 4 | 3 |
|  | Caudal communicating artery | CTA | 4 | 4 | 4 | 4 | 4 |
|  |  | TRICKS | 3 | 3 | 4 | 4 | 3 |
|  | Internal carotid artery | CTA | 4 | 4 | 4 | 4 | 4 |
|  |  | TRICKS | 4 | 4 | 4 | 4 | 3 |
|  | Dorsal sagittal sinus | CTA | 3 | 3 | 4 | 3 | 4 |
|  |  | TRICKS | 4 | 4 | 4 | 4 | 3 |
|  | Transverse sinus | CTA | 3 | 3 | 4 | 2 | 3 |
|  |  | TRICKS | 4 | 4 | 4 | 4 | 3 |
|  | Temporal sinus | CTA | 3 | 3 | 4 | 2 | 3 |
|  |  | TRICKS | 3 | 3 | 4 | 2 | 3 |
|  | Sigmoid sinus | CTA | 3 | 2 | 4 | 3 | 3 |
|  |  | TRICKS | 4 | 4 | 3 | 4 | 3 |
|  | Straight sinus | CTA | 3 | 3 | 3 | 3 | 4 |
|  |  | TRICKS | 4 | 4 | 4 | 4 | 3 |
|  | Dorsal cerebral vein | CTA | 2 | 2 | 2 | 1 | 3 |
|  |  | TRICKS | 3 | 4 | 4 | 4 | 3 |
|  | Infraorbital artery | CTA | 4 | 4 | 4 | 4 | 4 |
|  |  | TRICKS | 3 | 4 | 4 | 4 | 3 |
|  | Descending palatine artery | CTA | 4 | 4 | 4 | 4 | 4 |
|  |  | TRICKS | 3 | 4 | 4 | 4 | 3 |
|  | Maxillary artery | CTA | 4 | 4 | 4 | 4 | 4 |
|  |  | TRICKS | 4 | 4 | 4 | 4 | 3 |
|  | Caudal deep temporal artery | CTA | 4 | 3 | 3 | 4 | 4 |
|  |  | TRICKS | 3 | 2 | 3 | 4 | 3 |
|  | Caudal auricular artery | CTA | 4 | 4 | 4 | 4 | 4 |
|  |  | TRICKS | 4 | 4 | 4 | 4 | 3 |
|  | Maxillary vein | CTA | 4 | 4 | 4 | 4 | 4 |
|  |  | TRICKS | 4 | 4 | 4 | 4 | 3 |
|  | Superficial temporal vein | CTA | 4 | 4 | 4 | 4 | 4 |
|  |  | TRICKS | 4 | 4 | 4 | 4 | 3 |
|  | Dorsal external ophthalmic vein | CTA | 4 | 4 | 4 | 4 | 4 |
|  |  | TRICKS | 4 | 4 | 4 | 4 | 3 |
|  |  |  |  |  |  |  |  |
| Patient | 4 |  |  |  |  |  |  |
|  |  |  |  |  |  |  |  |
|  |  |  | Vascular visibility | Vascular delineation | Vascular connectivity | Vascular artifact associated adj. bone | Image quality |
|  | Basilar artery | CTA | 4 | 4 | 4 | 4 | 4 |
|  |  | TRICKS | 3 | 4 | 4 | 4 | 3 |
|  | Rostral cerebral artery | CTA | 4 | 4 | 4 | 4 | 4 |
|  |  | TRICKS | 3 | 3 | 3 | 4 | 3 |
|  | Middle cerebral artery | CTA | 4 | 4 | 4 | 4 | 4 |
|  |  | TRICKS | 4 | 4 | 4 | 4 | 3 |
|  | Caudal cerebral artery | CTA | 4 | 4 | 4 | 4 | 4 |
|  |  | TRICKS | 3 | 3 | 3 | 4 | 3 |
|  | Rostral cerebellar artery | CTA | 3 | 3 | 4 | 4 | 4 |
|  |  | TRICKS | 2 | 2 | 2 | 4 | 3 |
|  | Caudal communicating artery | CTA | 4 | 4 | 4 | 4 | 4 |
|  |  | TRICKS | 4 | 4 | 4 | 4 | 3 |
|  | Internal carotid artery | CTA | 4 | 4 | 4 | 4 | 4 |
|  |  | TRICKS | 4 | 4 | 4 | 4 | 3 |
|  | Dorsal sagittal sinus | CTA | 2 | 2 | 3 | 3 | 4 |
|  |  | TRICKS | 3 | 3 | 4 | 4 | 3 |
|  | Transverse sinus | CTA | 4 | 3 | 4 | 3 | 4 |
|  |  | TRICKS | 4 | 4 | 4 | 4 | 3 |
|  | Temporal sinus | CTA | 4 | 3 | 4 | 3 | 4 |
|  |  | TRICKS | 4 | 4 | 4 | 4 | 3 |
|  | Sigmoid sinus | CTA | 3 | 2 | 3 | 3 | 4 |
|  |  | TRICKS | 3 | 4 | 3 | 4 | 3 |
|  | Straight sinus | CTA | 2 | 2 | 3 | 3 | 4 |
|  |  | TRICKS | 3 | 4 | 4 | 4 | 3 |
|  | Dorsal cerebral vein | CTA | 2 | 2 | 2 | 1 | 4 |
|  |  | TRICKS | 4 | 4 | 4 | 4 | 3 |
|  | Infraorbital artery | CTA | 4 | 4 | 4 | 4 | 4 |
|  |  | TRICKS | 3 | 3 | 4 | 4 | 3 |
|  | Descending palatine artery | CTA | 4 | 4 | 4 | 4 | 4 |
|  |  | TRICKS | 3 | 3 | 4 | 4 | 3 |
|  | Maxillary artery | CTA | 4 | 4 | 4 | 4 | 4 |
|  |  | TRICKS | 3 | 3 | 4 | 4 | 3 |
|  | Caudal deep temporal artery | CTA | 4 | 4 | 4 | 4 | 4 |
|  |  | TRICKS | 3 | 3 | 4 | 4 | 3 |
|  | Caudal auricular artery | CTA | 4 | 4 | 4 | 4 | 4 |
|  |  | TRICKS | 3 | 3 | 3 | 4 | 3 |
|  | Maxillary vein | CTA | 4 | 4 | 4 | 4 | 4 |
|  |  | TRICKS | 4 | 4 | 4 | 4 | 3 |
|  | Superficial temporal vein | CTA | 3 | 4 | 3 | 4 | 4 |
|  |  | TRICKS | 3 | 4 | 3 | 4 | 3 |
|  | Dorsal external ophthalmic vein | CTA | 4 | 4 | 4 | 4 | 4 |
|  |  | TRICKS | 4 | 4 | 4 | 4 | 3 |
|  |  |  |  |  |  |  |  |
| Patient | 5 |  |  |  |  |  |  |
|  |  |  |  |  |  |  |  |
|  |  |  | Vascular visibility | Vascular delineation | Vascular connectivity | Vascular artifact associated adj. bone | Image quality |
|  | Basilar artery | CTA | 4 | 4 | 3 | 3 | 4 |
|  |  | TRICKS | 4 | 4 | 4 | 4 | 3 |
|  | Rostral cerebral artery | CTA | 4 | 4 | 4 | 4 | 4 |
|  |  | TRICKS | 4 | 4 | 4 | 4 | 3 |
|  | Middle cerebral artery | CTA | 4 | 4 | 4 | 4 | 4 |
|  |  | TRICKS | 3 | 4 | 3 | 4 | 3 |
|  | Caudal cerebral artery | CTA | 4 | 4 | 4 | 4 | 4 |
|  |  | TRICKS | 4 | 4 | 4 | 4 | 3 |
|  | Rostral cerebellar artery | CTA | 3 | 4 | 4 | 4 | 4 |
|  |  | TRICKS | 3 | 4 | 4 | 4 | 3 |
|  | Caudal communicating artery | CTA | 4 | 4 | 4 | 4 | 4 |
|  |  | TRICKS | 3 | 4 | 3 | 4 | 3 |
|  | Internal carotid artery | CTA | 4 | 3 | 3 | 3 | 4 |
|  |  | TRICKS | 3 | 3 | 3 | 4 | 3 |
|  | Dorsal sagittal sinus | CTA | 3 | 3 | 3 | 3 | 4 |
|  |  | TRICKS | 3 | 3 | 4 | 4 | 3 |
|  | Transverse sinus | CTA | 2 | 2 | 2 | 1 | 4 |
|  |  | TRICKS | 4 | 4 | 4 | 4 | 3 |
|  | Temporal sinus | CTA | 2 | 2 | 2 | 1 | 4 |
|  |  | TRICKS | 4 | 4 | 4 | 4 | 3 |
|  | Sigmoid sinus | CTA | 3 | 2 | 3 | 3 | 4 |
|  |  | TRICKS | 3 | 4 | 4 | 4 | 3 |
|  | Straight sinus | CTA | 2 | 2 | 3 | 1 | 4 |
|  |  | TRICKS | 4 | 4 | 4 | 4 | 3 |
|  | Dorsal cerebral vein | CTA | 1 | 1 | 1 | 1 | 4 |
|  |  | TRICKS | 3 | 4 | 4 | 4 | 3 |
|  | Infraorbital artery | CTA | 4 | 4 | 4 | 4 | 4 |
|  |  | TRICKS | 4 | 4 | 4 | 4 | 3 |
|  | Descending palatine artery | CTA | 4 | 4 | 4 | 4 | 4 |
|  |  | TRICKS | 4 | 4 | 4 | 4 | 3 |
|  | Maxillary artery | CTA | 4 | 4 | 3 | 3 | 4 |
|  |  | TRICKS | 4 | 4 | 4 | 4 | 3 |
|  | Caudal deep temporal artery | CTA | 4 | 4 | 4 | 3 | 4 |
|  |  | TRICKS | 3 | 4 | 4 | 4 | 3 |
|  | Caudal auricular artery | CTA | 4 | 4 | 4 | 4 | 4 |
|  |  | TRICKS | 3 | 3 | 4 | 4 | 3 |
|  | Maxillary vein | CTA | 4 | 4 | 4 | 4 | 4 |
|  |  | TRICKS | 3 | 4 | 4 | 4 | 3 |
|  | Superficial temporal vein | CTA | 4 | 4 | 4 | 4 | 4 |
|  |  | TRICKS | 3 | 4 | 4 | 4 | 3 |
|  | Dorsal external ophthalmic vein | CTA | 4 | 4 | 4 | 3 | 4 |
|  |  | TRICKS | 4 | 4 | 3 | 4 | 3 |
|  |  |  |  |  |  |  |  |
| Patient | 6 |  |  |  |  |  |  |
|  |  |  |  |  |  |  |  |
|  |  |  | Vascular visibility | Vascular delineation | Vascular connectivity | Vascular artifact associated adj. bone | Image quality |
|  | Basilar artery | CTA | 4 | 4 | 4 | 4 | 4 |
|  |  | TRICKS | 4 | 4 | 4 | 4 | 3 |
|  | Rostral cerebral artery | CTA | 4 | 4 | 4 | 4 | 4 |
|  |  | TRICKS | 3 | 3 | 4 | 4 | 3 |
|  | Middle cerebral artery | CTA | 4 | 4 | 4 | 4 | 4 |
|  |  | TRICKS | 3 | 3 | 4 | 4 | 3 |
|  | Caudal cerebral artery | CTA | 4 | 4 | 4 | 4 | 4 |
|  |  | TRICKS | 3 | 3 | 4 | 4 | 3 |
|  | Rostral cerebellar artery | CTA | 4 | 4 | 4 | 4 | 4 |
|  |  | TRICKS | 3 | 3 | 4 | 4 | 3 |
|  | Caudal communicating artery | CTA | 4 | 4 | 4 | 4 | 4 |
|  |  | TRICKS | 3 | 4 | 4 | 4 | 3 |
|  | Internal carotid artery | CTA | 4 | 4 | 4 | 4 | 4 |
|  |  | TRICKS | 4 | 3 | 4 | 4 | 3 |
|  | Dorsal sagittal sinus | CTA | 3 | 2 | 4 | 3 | 4 |
|  |  | TRICKS | 3 | 3 | 4 | 4 | 3 |
|  | Transverse sinus | CTA | 4 | 3 | 4 | 3 | 4 |
|  |  | TRICKS | 4 | 4 | 4 | 4 | 3 |
|  | Temporal sinus | CTA | 4 | 3 | 3 | 3 | 3 |
|  |  | TRICKS | 4 | 4 | 4 | 4 | 3 |
|  | Sigmoid sinus | CTA | 3 | 3 | 4 | 4 | 4 |
|  |  | TRICKS | 4 | 4 | 4 | 4 | 3 |
|  | Straight sinus | CTA | 4 | 3 | 4 | 4 | 4 |
|  |  | TRICKS | 3 | 4 | 4 | 4 | 3 |
|  | Dorsal cerebral vein | CTA | 4 | 4 | 4 | 3 | 4 |
|  |  | TRICKS | 3 | 3 | 4 | 4 | 3 |
|  | Infraorbital artery | CTA | 4 | 4 | 4 | 4 | 4 |
|  |  | TRICKS | 2 | 2 | 4 | 4 | 3 |
|  | Descending palatine artery | CTA | 4 | 4 | 4 | 4 | 4 |
|  |  | TRICKS | 3 | 2 | 4 | 4 | 3 |
|  | Maxillary artery | CTA | 4 | 4 | 4 | 4 | 4 |
|  |  | TRICKS | 3 | 2 | 4 | 4 | 3 |
|  | Caudal deep temporal artery | CTA | 3 | 4 | 4 | 4 | 4 |
|  |  | TRICKS | 2 | 2 | 4 | 4 | 3 |
|  | Caudal auricular artery | CTA | 4 | 4 | 4 | 4 | 4 |
|  |  | TRICKS | 3 | 2 | 4 | 4 | 3 |
|  | Maxillary vein | CTA | 4 | 4 | 4 | 4 | 4 |
|  |  | TRICKS | 2 | 2 | 4 | 4 | 3 |
|  | Superficial temporal vein | CTA | 4 | 4 | 4 | 4 | 4 |
|  |  | TRICKS | 3 | 3 | 4 | 4 | 3 |
|  | Dorsal external ophthalmic vein | CTA | 4 | 4 | 4 | 4 | 4 |
|  |  | TRICKS | 4 | 4 | 4 | 4 | 3 |
|  |  |  |  |  |  |  |  |
| Patient | 7 |  |  |  |  |  |  |
|  |  |  |  |  |  |  |  |
|  |  |  | Vascular visibility | Vascular delineation | Vascular connectivity | Vascular artifact associated adj. bone | Image quality |
|  | Basilar artery | CTA | 4 | 4 | 4 | 4 | 4 |
|  |  | TRICKS | 4 | 4 | 4 | 4 | 3 |
|  | Rostral cerebral artery | CTA | 4 | 4 | 4 | 4 | 4 |
|  |  | TRICKS | 3 | 3 | 3 | 4 | 3 |
|  | Middle cerebral artery | CTA | 4 | 4 | 4 | 4 | 4 |
|  |  | TRICKS | 3 | 3 | 3 | 4 | 3 |
|  | Caudal cerebral artery | CTA | 4 | 4 | 4 | 4 | 4 |
|  |  | TRICKS | 3 | 4 | 4 | 4 | 3 |
|  | Rostral cerebellar artery | CTA | 4 | 4 | 4 | 4 | 4 |
|  |  | TRICKS | 3 | 3 | 3 | 4 | 3 |
|  | Caudal communicating artery | CTA | 4 | 4 | 4 | 4 | 4 |
|  |  | TRICKS | 4 | 3 | 3 | 4 | 3 |
|  | Internal carotid artery | CTA | 4 | 4 | 4 | 4 | 4 |
|  |  | TRICKS | 4 | 4 | 4 | 4 | 3 |
|  | Dorsal sagittal sinus | CTA | 3 | 4 | 4 | 4 | 4 |
|  |  | TRICKS | 4 | 4 | 4 | 4 | 3 |
|  | Transverse sinus | CTA | 3 | 3 | 4 | 3 | 4 |
|  |  | TRICKS | 4 | 4 | 4 | 4 | 3 |
|  | Temporal sinus | CTA | 3 | 3 | 4 | 4 | 4 |
|  |  | TRICKS | 4 | 4 | 4 | 4 | 3 |
|  | Sigmoid sinus | CTA | 3 | 3 | 4 | 3 | 3 |
|  |  | TRICKS | 4 | 4 | 4 | 4 | 3 |
|  | Straight sinus | CTA | 3 | 3 | 3 | 3 | 4 |
|  |  | TRICKS | 3 | 3 | 3 | 4 | 3 |
|  | Dorsal cerebral vein | CTA | 4 | 3 | 3 | 3 | 4 |
|  |  | TRICKS | 4 | 4 | 4 | 4 | 3 |
|  | Infraorbital artery | CTA | 4 | 4 | 4 | 4 | 4 |
|  |  | TRICKS | 3 | 4 | 4 | 4 | 3 |
|  | Descending palatine artery | CTA | 4 | 4 | 4 | 4 | 4 |
|  |  | TRICKS | 3 | 3 | 4 | 4 | 3 |
|  | Maxillary artery | CTA | 4 | 4 | 4 | 4 | 4 |
|  |  | TRICKS | 3 | 4 | 4 | 4 | 3 |
|  | Caudal deep temporal artery | CTA | 4 | 4 | 4 | 4 | 4 |
|  |  | TRICKS | 3 | 3 | 4 | 4 | 3 |
|  | Caudal auricular artery | CTA | 4 | 4 | 4 | 4 | 4 |
|  |  | TRICKS | 3 | 4 | 4 | 4 | 3 |
|  | Maxillary vein | CTA | 4 | 4 | 4 | 4 | 4 |
|  |  | TRICKS | 4 | 4 | 4 | 4 | 3 |
|  | Superficial temporal vein | CTA | 4 | 4 | 4 | 4 | 4 |
|  |  | TRICKS | 4 | 4 | 3 | 4 | 3 |
|  | Dorsal external ophthalmic vein | CTA | 4 | 4 | 4 | 4 | 4 |
|  |  | TRICKS | 4 | 4 | 4 | 4 | 3 |
|  |  |  |  |  |  |  |  |
| Patient | 8 |  |  |  |  |  |  |
|  |  |  |  |  |  |  |  |
|  |  |  | Vascular visibility | Vascular delineation | Vascular connectivity | Vascular artifact associated adj. bone | Image quality |
|  | Basilar artery | CTA | 4 | 4 | 4 | 4 | 4 |
|  |  | TRICKS | 3 | 4 | 4 | 4 | 3 |
|  | Rostral cerebral artery | CTA | 3 | 4 | 4 | 4 | 4 |
|  |  | TRICKS | 4 | 3 | 4 | 4 | 3 |
|  | Middle cerebral artery | CTA | 4 | 4 | 4 | 4 | 4 |
|  |  | TRICKS | 4 | 4 | 4 | 4 | 3 |
|  | Caudal cerebral artery | CTA | 4 | 4 | 4 | 4 | 4 |
|  |  | TRICKS | 3 | 4 | 4 | 4 | 3 |
|  | Rostral cerebellar artery | CTA | 4 | 4 | 4 | 4 | 4 |
|  |  | TRICKS | 3 | 4 | 4 | 4 | 3 |
|  | Caudal communicating artery | CTA | 4 | 4 | 4 | 4 | 4 |
|  |  | TRICKS | 4 | 4 | 4 | 4 | 3 |
|  | Internal carotid artery | CTA | 4 | 4 | 3 | 3 | 4 |
|  |  | TRICKS | 4 | 4 | 4 | 4 | 3 |
|  | Dorsal sagittal sinus | CTA | 3 | 3 | 4 | 3 | 4 |
|  |  | TRICKS | 4 | 4 | 4 | 4 | 3 |
|  | Transverse sinus | CTA | 3 | 3 | 4 | 3 | 4 |
|  |  | TRICKS | 4 | 4 | 4 | 4 | 3 |
|  | Temporal sinus | CTA | 4 | 3 | 4 | 4 | 4 |
|  |  | TRICKS | 4 | 4 | 4 | 4 | 3 |
|  | Sigmoid sinus | CTA | 4 | 4 | 3 | 3 | 4 |
|  |  | TRICKS | 3 | 4 | 4 | 4 | 3 |
|  | Straight sinus | CTA | 4 | 4 | 4 | 4 | 4 |
|  |  | TRICKS | 4 | 4 | 4 | 4 | 3 |
|  | Dorsal cerebral vein | CTA | 4 | 4 | 4 | 4 | 4 |
|  |  | TRICKS | 3 | 4 | 4 | 4 | 3 |
|  | Infraorbital artery | CTA | 4 | 4 | 4 | 4 | 4 |
|  |  | TRICKS | 3 | 4 | 4 | 4 | 3 |
|  | Descending palatine artery | CTA | 4 | 4 | 4 | 4 | 4 |
|  |  | TRICKS | 3 | 4 | 4 | 4 | 3 |
|  | Maxillary artery | CTA | 4 | 4 | 4 | 4 | 4 |
|  |  | TRICKS | 4 | 4 | 4 | 4 | 3 |
|  | Caudal deep temporal artery | CTA | 4 | 4 | 3 | 3 | 4 |
|  |  | TRICKS | 4 | 4 | 4 | 4 | 3 |
|  | Caudal auricular artery | CTA | 4 | 4 | 4 | 4 | 4 |
|  |  | TRICKS | 4 | 4 | 4 | 4 | 3 |
|  | Maxillary vein | CTA | 4 | 4 | 4 | 4 | 4 |
|  |  | TRICKS | 4 | 4 | 4 | 4 | 3 |
|  | Superficial temporal vein | CTA | 4 | 4 | 4 | 4 | 4 |
|  |  | TRICKS | 4 | 4 | 3 | 4 | 4 |
|  | Dorsal external ophthalmic vein | CTA | 4 | 4 | 4 | 4 | 4 |
|  |  | TRICKS | 4 | 4 | 4 | 4 | 3 |
|  |  |  |  |  |  |  |  |
| Patient | 9 |  |  |  |  |  |  |
|  |  |  |  |  |  |  |  |
|  |  |  | Vascular visibility | Vascular delineation | Vascular connectivity | Vascular artifact associated adj. bone | Image quality |
|  | Basilar artery | CTA | 4 | 4 | 4 | 4 | 4 |
|  |  | TRICKS | 3 | 3 | 4 | 4 | 3 |
|  | Rostral cerebral artery | CTA | 4 | 4 | 3 | 4 | 4 |
|  |  | TRICKS | 3 | 3 | 3 | 4 | 3 |
|  | Middle cerebral artery | CTA | 4 | 4 | 4 | 4 | 4 |
|  |  | TRICKS | 3 | 4 | 4 | 4 | 3 |
|  | Caudal cerebral artery | CTA | 4 | 4 | 4 | 4 | 4 |
|  |  | TRICKS | 3 | 2 | 4 | 4 | 3 |
|  | Rostral cerebellar artery | CTA | 4 | 4 | 4 | 4 | 4 |
|  |  | TRICKS | 2 | 2 | 2 | 4 | 3 |
|  | Caudal communicating artery | CTA | 4 | 4 | 4 | 4 | 4 |
|  |  | TRICKS | 2 | 2 | 2 | 4 | 3 |
|  | Internal carotid artery | CTA | 4 | 3 | 3 | 3 | 4 |
|  |  | TRICKS | 3 | 3 | 3 | 4 | 3 |
|  | Dorsal sagittal sinus | CTA | 3 | 2 | 3 | 2 | 4 |
|  |  | TRICKS | 3 | 3 | 4 | 4 | 3 |
|  | Transverse sinus | CTA | 3 | 2 | 3 | 3 | 4 |
|  |  | TRICKS | 4 | 4 | 4 | 4 | 3 |
|  | Temporal sinus | CTA | 3 | 2 | 2 | 2 | 4 |
|  |  | TRICKS | 4 | 4 | 4 | 4 | 3 |
|  | Sigmoid sinus | CTA | 3 | 2 | 3 | 3 | 4 |
|  |  | TRICKS | 3 | 2 | 3 | 4 | 3 |
|  | Straight sinus | CTA | 3 | 3 | 3 | 3 | 4 |
|  |  | TRICKS | 3 | 3 | 4 | 4 | 3 |
|  | Dorsal cerebral vein | CTA | 3 | 2 | 2 | 2 | 4 |
|  |  | TRICKS | 3 | 3 | 4 | 4 | 3 |
|  | Infraorbital artery | CTA | 4 | 4 | 4 | 4 | 4 |
|  |  | TRICKS | 3 | 3 | 4 | 4 | 3 |
|  | Descending palatine artery | CTA | 3 | 4 | 4 | 4 | 4 |
|  |  | TRICKS | 3 | 3 | 4 | 4 | 3 |
|  | Maxillary artery | CTA | 4 | 4 | 4 | 4 | 4 |
|  |  | TRICKS | 3 | 3 | 4 | 4 | 2 |
|  | Caudal deep temporal artery | CTA | 4 | 4 | 4 | 4 | 4 |
|  |  | TRICKS | 2 | 2 | 3 | 4 | 3 |
|  | Caudal auricular artery | CTA | 4 | 4 | 4 | 4 | 4 |
|  |  | TRICKS | 2 | 2 | 3 | 4 | 2 |
|  | Maxillary vein | CTA | 4 | 4 | 4 | 4 | 4 |
|  |  | TRICKS | 4 | 4 | 4 | 4 | 3 |
|  | Superficial temporal vein | CTA | 3 | 3 | 3 | 4 | 4 |
|  |  | TRICKS | 3 | 3 | 4 | 4 | 3 |
|  | Dorsal external ophthalmic vein | CTA | 3 | 3 | 4 | 4 | 3 |
|  |  | TRICKS | 4 | 4 | 4 | 4 | 3 |
|  |  |  |  |  |  |  |  |
| Patient | 10 |  |  |  |  |  |  |
|  |  |  | Vascular visibility | Vascular delineation | Vascular connectivity | Vascular artifact associated adj. bone | Image quality |
|  | Basilar artery | CTA | 4 | 4 | 4 | 4 | 4 |
|  |  | TRICKS | 3 | 3 | 4 | 4 | 3 |
|  | Rostral cerebral artery | CTA | 4 | 4 | 4 | 4 | 4 |
|  |  | TRICKS | 3 | 3 | 4 | 4 | 3 |
|  | Middle cerebral artery | CTA | 4 | 4 | 4 | 4 | 4 |
|  |  | TRICKS | 3 | 3 | 4 | 4 | 3 |
|  | Caudal cerebral artery | CTA | 4 | 4 | 4 | 4 | 4 |
|  |  | TRICKS | 3 | 3 | 3 | 4 | 3 |
|  | Rostral cerebellar artery | CTA | 4 | 4 | 4 | 4 | 4 |
|  |  | TRICKS | 3 | 3 | 3 | 4 | 3 |
|  | Caudal communicating artery | CTA | 4 | 4 | 4 | 4 | 4 |
|  |  | TRICKS | 2 | 3 | 3 | 4 | 3 |
|  | Internal carotid artery | CTA | 4 | 4 | 4 | 4 | 4 |
|  |  | TRICKS | 4 | 4 | 4 | 4 | 3 |
|  | Dorsal sagittal sinus | CTA | 4 | 4 | 4 | 4 | 4 |
|  |  | TRICKS | 4 | 4 | 4 | 4 | 3 |
|  | Transverse sinus | CTA | 4 | 4 | 4 | 4 | 4 |
|  |  | TRICKS | 4 | 4 | 4 | 4 | 3 |
|  | Temporal sinus | CTA | 4 | 4 | 4 | 4 | 4 |
|  |  | TRICKS | 4 | 4 | 4 | 4 | 3 |
|  | Sigmoid sinus | CTA | 3 | 4 | 4 | 3 | 4 |
|  |  | TRICKS | 3 | 4 | 4 | 4 | 3 |
|  | Straight sinus | CTA | 4 | 4 | 4 | 4 | 4 |
|  |  | TRICKS | 3 | 4 | 4 | 4 | 3 |
|  | Dorsal cerebral vein | CTA | 4 | 4 | 4 | 4 | 4 |
|  |  | TRICKS | 4 | 4 | 4 | 4 | 3 |
|  | Infraorbital artery | CTA | 4 | 4 | 4 | 4 | 4 |
|  |  | TRICKS | 4 | 4 | 4 | 4 | 3 |
|  | Descending palatine artery | CTA | 4 | 4 | 4 | 4 | 4 |
|  |  | TRICKS | 3 | 4 | 4 | 4 | 3 |
|  | Maxillary artery | CTA | 4 | 4 | 4 | 4 | 4 |
|  |  | TRICKS | 4 | 4 | 4 | 4 | 3 |
|  | Caudal deep temporal artery | CTA | 4 | 4 | 4 | 4 | 4 |
|  |  | TRICKS | 3 | 4 | 4 | 4 | 3 |
|  | Caudal auricular artery | CTA | 4 | 4 | 4 | 4 | 4 |
|  |  | TRICKS | 3 | 4 | 4 | 4 | 3 |
|  | Maxillary vein | CTA | 4 | 4 | 4 | 4 | 4 |
|  |  | TRICKS | 4 | 4 | 4 | 4 | 3 |
|  | Superficial temporal vein | CTA | 4 | 4 | 4 | 4 | 4 |
|  |  | TRICKS | 4 | 4 | 4 | 4 | 3 |
|  | Dorsal external ophthalmic vein | CTA | 4 | 4 | 4 | 4 | 4 |
|  |  | TRICKS | 4 | 4 | 4 | 4 | 3 |
